# Evolutionary rescue in a host–pathogen system results in coexistence not clearance

**DOI:** 10.1111/eva.12568

**Published:** 2017-11-20

**Authors:** Mark Redpath Christie, Catherine Laura Searle

**Affiliations:** ^1^ Department of Biological Sciences Purdue University West Lafayette IN USA; ^2^ Department of Forestry and Natural Resources Purdue University West Lafayette IN USA

**Keywords:** *Batrachochytrium dendrobatidis*, chytridiomycosis, emerging infectious disease, extinction, host–parasite interactions, rapid evolution, rapid genetic adaptation, susceptibility

## Abstract

The evolutionary rescue of host populations may prevent extinction from novel pathogens. However, the conditions that facilitate rapid evolution of hosts, in particular the population variation in host susceptibility, and the effects of host evolution in response to pathogens on population outcomes remain largely unknown. We constructed an individual‐based model to determine the relationships between genetic variation in host susceptibility and population persistence in an amphibian‐fungal pathogen (*Batrachochytrium dendrobatidis*) system. We found that host populations can rapidly evolve reduced susceptibility to a novel pathogen and that this rapid evolution led to a 71‐fold increase in the likelihood of host–pathogen coexistence. However, the increased rates of coexistence came at a cost to host populations; fewer populations cleared infection, population sizes were depressed, and neutral genetic diversity was lost. Larger adult host population sizes and greater adaptive genetic variation prior to the onset of pathogen introduction led to substantially reduced rates of extinction, suggesting that populations with these characteristics should be prioritized for conservation when species are threatened by novel infectious diseases.

## 1 INTRODUCTION

Infectious diseases are rapidly becoming a principal threat to biodiversity (Daszak, 2000; Smith, Sax, & Lafferty, 2006). Myriad factors such as global climate change and habitat fragmentation are intensifying disease outbreaks in natural systems, and the outcomes can be severe (Aguirre & Tabor, 2008; Harvell et al., 2002). Treatments for infected populations, if available, are often cost prohibitive, logistically intractable or result in substantial side effects to nontarget species (*e.g.,* Bosch et al., 2015; Pederson & Fenton, 2015). One effective preventative measure could be to maintain genetic diversity within natural populations such that infected host populations can quickly respond to the selective pressures imposed by disease outbreaks. However, the genetic, demographic and environmental conditions that allow host populations to rapidly evolve in response to a pathogen remain largely unknown. Understanding the evolutionary processes that affect disease dynamics on ecologically relevant timescales is essential for identifying which populations are at the greatest risk for disease‐driven declines and for predicting the long‐term persistence of host populations.

From a mechanistic perspective, host populations can respond to infectious disease by evolving resistance or tolerance to the pathogen (reviewed in Altizer, Harvell, & Friedle, 2003; Penczykowski, Forde, & Duffy, 2011). Recent empirical work has revealed that evolutionary responses can happen very quickly, sometimes within only a handful of generations (Christie, Marine, Fox, French, & Blouin, 2016; Hendry, 2016; Stockwell, Hendry, & Kinnison, 2003). When an environmental change impacts a population, a rapid response to selection can prevent population extinction in a process known as “evolutionary rescue” (Carlson, Cunningham, & Westley, 2014). Although much of evolutionary rescue research has focused on abiotic environmental changes (e.g., climate change, pollution; reviewed in Gonzalez, Ronce, Ferriere, & Hochberg, 2012; Carlson et al., 2014), biotic perturbations, such as the introduction of a novel pathogen, likely have similar outcomes. However, the introduction of a pathogen is different from abiotic stress because the effects of pathogens on host populations are often density‐dependent (e.g., for many pathogens with direct transmission). Therefore, the selective pressures from a pathogen will change depending on the density, abundance and susceptibility of the host population (e.g., Burdon & Chilvers, 1982; Duffy et al., 2012). Furthermore, evolutionary rescue could result in two possible outcomes in disease systems, host–pathogen coexistence or clearance (pathogen extinction), and it is unclear under what conditions and how frequently each of these two outcomes will occur. Thus, there is a need to understand evolutionary rescue in host–pathogen systems (Bonte, Hovestadt, & Poetke, 2009; Gandon, Hochberg, Holt, & Day, 2012), particularly when populations are confronted with novel or introduced pathogens.

One parameter that is critical for improving our understanding of evolutionary rescue in host–pathogen systems is the amount and type of genetic variation present within host populations. Some disease models predict that increased host genetic variation has little effect on the spread of pathogens within populations (Nath, Woolliams, & Bishop, 2008; Springbett, MacKenzie, Woolliams, & Bishop, 2003; Yates, Antia, & Regoes, 2006), while others show that high genetic variation can directly reduce disease spread (Lively, 2010). However, the majority of experimental studies investigating the effects of genetic variation in host susceptibility have used single generations and have not allowed host evolution to occur (e.g., Zhu et al., 2000; Hughes & Boomsma, 2004; but see Altermatt & Ebert, 2008). Thus, while host genetic variation can play a significant role in short‐term disease dynamics (i.e., single host generations), much remains unknown regarding the relationship between variation in host susceptibility and the ability of host populations to evolve over multiple generations. For example, it is unknown how the within‐population distribution of genetically based host susceptibilities interacts to influence the likelihood of evolutionary rescue. Additionally, rapid host evolution may fail to rescue populations from going extinct in some scenarios (i.e., there are limits to evolutionary rescue; Bell, 2013; Osmond & Mazancourt, 2013; Stewart et al., 2017). Thus, more studies are necessary to understand the conditions that allow for evolutionary rescue in response to pathogens.

One pathogen that has had large negative effects on host populations is the fungus, *Batrachochytrium dendrobatidis* (*Bd*), which infects the keratinized structures of amphibians and can result in high rates of mortality (Garner et al., 2009; Searle, & Gervasi, et al., 2011). Global amphibian population declines and extinctions have been directly linked to the introduction of this pathogen (Skerratt et al., 2007). However, some amphibian species and populations are seemingly unaffected by *Bd*, suffering no detectable population declines in its presence (e.g., Daszak et al., 2004, 2005; Reeder, Pessier, & Vredenburg, 2012). Variation in disease dynamics after *Bd* introduction may be driven by a number of variables including rapid evolution of amphibian hosts in response to *Bd*. There is evidence that *Bd* epidemics can impose directional selection for MHC genes incurring resistance (Bataille et al., 2015; Savage & Zamudio, 2011, 2016) and individuals from populations with a history of exposure to *Bd* can experience reduced pathogen loads compared to individuals from naïve populations (Knapp et al., 2016). One recent study in toads found that estimates of narrow‐sense heritability for *Bd*‐load were fairly substantial (*h* ^*2*^ = 0.22; Palomar, Bosch, & Cano, 2016), suggesting that there is heritable variation for susceptibility in natural populations and that these populations may be able to rapidly respond to the strong selection imposed by *Bd*. Critically, however, it remains unknown whether the evolution of amphibian hosts in response to *Bd* can lead to evolutionary rescue. Several previous models of amphibian‐*Bd* dynamics have been developed (Briggs, Knapp, & Vredenburg, 2010; Briggs, Vredenburg, Knapp, & Rachowicz, 2005; Converse et al., 2016; Drawert, Griesemer, Petzold, & Briggs, 2017; Grogan et al., 2016; Wilber, Langwig, Kilpatrick, McCallum, & Briggs, 2016), but none focuses on evolution of the amphibian host. The goals of our study were to concentrate on the amphibian‐*Bd* system to (i) identify how variation in host susceptibility (range and distribution of host susceptibilities) and host genetic diversity (number of host genotypes) affects the ability of populations to rapidly evolve in response to a pathogen and (ii) quantify how this host evolution affects population recovery (i.e., evolutionary rescue).

## 2 MATERIALS AND METHODS

We constructed a spatially explicit, forward‐time, individual‐based model of an amphibian population to determine the relationships between variation in host susceptibility, host evolution and population persistence after the introduction of *Bd*. We chose an individual‐based model because we could vary the susceptibility of each individual within a population (DeAngelis & Grimm, 2014), and manipulate a range of population and disease parameters (e.g., adult carrying capacity, transmission rates). Many of our response variables are at the population level because population‐level responses are often what is important from a conservation standpoint. However, selection in the model only occurs at the individual level. Our model followed life‐history characteristics consistent with a majority of temperate anuran species (Wells, 1977, 2007; Table S1).

### 2.1 Model overview

**Figure 1:**
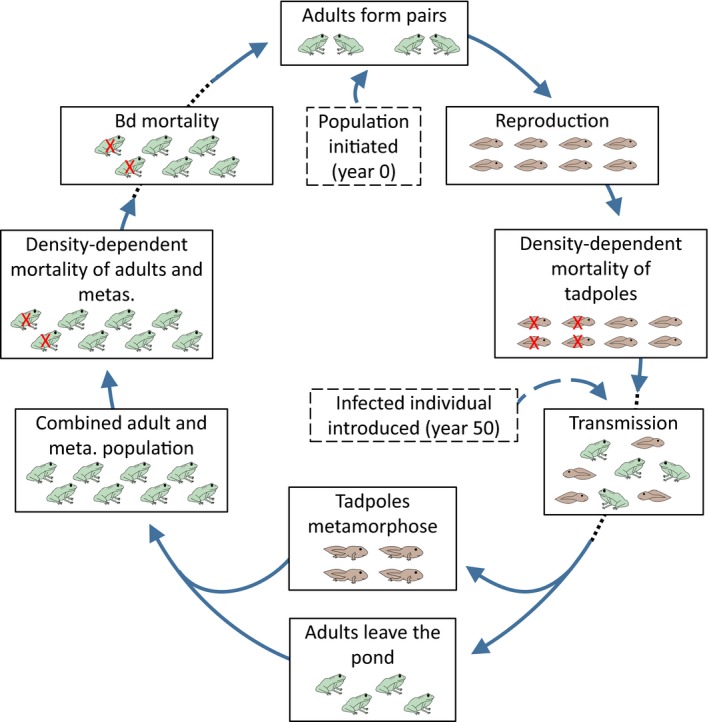
Schematic representation of the steps in our model. The population is initiated in the aquatic environment with adult population size at carrying capacity. Adults form pairs randomly and susceptibility to *Bd* has a heritable component that is passed on to the offspring. Reproduction is followed by density‐dependent mortality of tadpoles (based on tadpole carrying capacity) and transmission of *Bd*. The population then transitions to the terrestrial environment with tadpoles completing metamorphosis (becoming metamorphs: “metas.”) and adults leaving the pond. Density‐dependent adult mortality occurs (based on adult carrying capacity) followed by *Bd*‐associated mortality (based on individual genotype‐specific mortality; *GSM*), after which the population returns to the aquatic environment to begin a new year. A single infected individual is introduced in year 50; in years 1–49 both the transmission and *Bd* mortality steps are omitted

Our model consisted of a series of sequential steps (Figure 1), where each simulation began with the creation of two spatially discrete habitats: (i) an aquatic breeding habitat where adults congregated to breed and where tadpoles were born, and (ii) a terrestrial overwintering habitat, where individuals were solitary. We began with unrelated adults at a population size equal to their carrying capacity (*K*_*adults*_). Multilocus diploid genotypes for each individual were created in accordance with Hardy–Weinberg Equilibrium and consisted of 100 neutral bi‐allelic loci (i.e., single nucleotide polymorphisms; SNPs) with an initial minor allele frequency of 0.2. We also assigned a *Bd*‐susceptibility value for each individual, referred to as genotype‐specific mortality (*GSM*), which had values ranging from 0 to 1, indicating the probability that a genotype dies each year from *Bd* if infected. We parameterized the model based on existing literature (Table S1).

### 2.2 Reproduction

The first step in the model was reproduction, which occurred by randomly pairing all adults and creating a maximum of 100 offspring for each pair. As most offspring did not survive to adulthood, this procedure created high variance in reproductive success among pairs, which is commonly observed in natural populations (Christie, Marine, French, Waples, & Blouin, 2012; Hedrick, 2005). Varying the maximum number of offspring per pair from 10 to 1,000 resulted in little change to the number of offspring from each family that survive to metamorphosis (Fig. S1). After offspring were created, multilocus genotypes were assigned to each individual in accordance with Mendelian inheritance (i.e., the diploid offspring inherited one randomly selected allele from each parent). To assign *GSM* values for each offspring in a family, we sampled a random deviate from a normal distribution where the mean of the distribution was equal to the mid‐parent *GSM* value and the variance equal to half of the genetic variance of the initial population (e.g., Dunlop, Baskett, Heino, & Dieckmann, 2009; Dunlop, Heino, & Dieckmann 2009). Variation in phenotype driven by the environment would increase this variance.

### 2.3 Tadpole mortality

After reproduction, the offspring (tadpoles) moved randomly throughout the pond and density‐dependent mortality of tadpoles occurred based on tadpole carrying capacity (*K*_*tads*_). This mortality was random with respect to *GSM*, neutral genotype, parentage and location. High mortality at the tadpole stage is consistent with most temperate amphibians which have high fecundity and type III survivorship (e.g., Anderson, Hassinge, & Dalrymple, 1971; Calef, 1973).

### 2.4 Transmission

The model ran for 49 years in the absence of *Bd*, and at year 50, we introduced a single *Bd*‐infected adult. Because we employed an individual‐based model, keeping track of each zoospore shed by each host (up to 68 zoospores per minute; Reeder et al., 2012) was computationally intractable. Thus, we modelled transmission as a density‐dependent process by setting the maximum distance that a zoospore could travel to reach a neighbouring host (in a 100 × 100 m environment). We simulated a season of transmission where transmission occurred every hour throughout a three‐month period. Each hour, we (i) allowed all individuals of both stages to randomly move throughout the breeding habitat, (ii) stopped the model and recorded the position of all individuals in two‐dimensional space and (iii) changed the infection status for uninfected individuals that came in contact with an infected individual (i.e., within the maximum transmission distance). The size of the breeding habitat was kept constant, such that changes in abundance led to concomitant changes in density. We used a binary classification of individual infection status (infected/uninfected), rather than modelling infection loads because pathogen loads can vary greatly both between (Searle, Biga, Spatafora, & Blaustein, 2011; Venesky, Liu, Sauer, & Rohr, 2014) and within species (Grogan et al., 2016). However, we captured some variation in pathogen loads in our model because (i) pathogen loads and *Bd*‐induced mortality in the field are often greatest in the winter (Grogan et al., 2016; Phillott et al., 2013), simulated with *Bd*‐induced winter mortality in our model and (ii) newly infected individuals only transmitted *Bd* after they had been infected for a year, which would be expected if pathogen loads must accumulate before transmission. We chose to model transmission in a density‐dependent manner rather than through the environment because (i) previous studies have found evidence for density‐dependent transmission of *Bd* (Rachowicz & Briggs, 2007) and that altering the amount of *Bd* in the environment has little to no impact on host population outcomes (Drawert et al., 2017), (ii) in most conditions, *Bd* zoospores remain motile for only short periods of time outside the host (Piotrowski, Annis, & Longcore, 2004; Voyles et al., 2012; Woodhams, Alford, Briggs, Johnson, & Rollins‐Smith, 2008) and are unlikely to survive long periods of freezing temperatures in the winter (Boyle et al., 2003), and (iii) transmission and mortality are decoupled in this system, such that allowing for build‐up of zoospores within the environment would only influence the number of infected individuals within a given year, which we modelled by changing the transmission rate (see below). Once infected with *Bd*, individuals remained infected through metamorphosis and across years. Comparisons between our approach to modelling transmission and a probability‐based approach resulted in nearly identical results (Fig. S2).

### 2.5 Adult mortality

After the transmission step, tadpoles became metamorphs (newly metamorphosed individuals), both adults and metamorphs moved to the terrestrial environment, and density‐dependent mortality of the adults and metamorphs occurred based on adult carrying capacity (*K*_*adults*_). The number of adults was recorded at the beginning of each year in the model (immediately before reproduction) and mortality on metamorphs and adults occurred at the end of the year (after adult emigration) to keep the combined adult and metamorph population near *K*_*adults*_ in the absence of any *Bd*‐related mortality. Variation in density‐dependent mortality was introduced each year by sampling a random deviate from a normal distribution (μ = *N−N*_*t+1*_; *SD *= 7). All tadpoles completed metamorphosis in one season, which is common for the majority of temperate anurans (Wells, 2007; data from Bancroft et al., 2011).

### 2.6 *Bd* mortality

Once an individual became infected, its probability of dying from infection depended upon its *GSM* value. For example, individuals with *GSM* = 0 never died from infection, while individuals with *GSM* = 1 always died the first year they became infected. *GSM* values between 0 and 1 indicated the probability that infected individuals would die from infection each year (e.g., *GSM* = 0.80 indicated an 80% chance of dying each year if infected). We did not include any trade‐offs between *GSM* and other parameters (e.g., reproductive success) because, to date, there is no evidence that there is a cost to resistance or tolerance in the *Bd*‐amphibian system. Because the vast majority of anuran species appear to be tolerant of infection as tadpoles (e.g., Briggs et al., 2005; Garner et al., 2009), only infected adults and metamorphs died from *Bd* infection. After *Bd*‐related mortality, metamorphs became adults and we recorded the number of infected and uninfected individuals, genetic diversity and the distribution of *GSM* values. Finally, the model year was incremented and the next year began with reproduction.

### 2.7 Population outcomes

For each simulation, the population was monitored until it either went extinct or 100 years elapsed after *Bd* introduction (150 years total) at which point the population outcome was recorded. Populations were classified as “extinct” if there were no remaining amphibians, “cleared” if only uninfected amphibians remained (i.e., the population cleared infection), or “coexisting” if infected individuals were present in the population (i.e., both the host and pathogen were present). All model simulations and analyses were run in R version 3.2 (R Core Team 2016).

### 2.8 Fixed within‐population susceptibility

We first modelled scenarios where all adults and offspring within a population had the same *GSM*, such that host evolution could not occur. This lack of variation is likely present in some populations (e.g., those with genetic constraints or no additive genetic variation) and allowed us to systematically examine the interactions between *GSM*, population demography and transmission on population outcome. We varied *GSM* from 0 to 1 (by increments of 0.1), *K*_*adults*_ as 100, 200 or 300 individuals, and transmission from low to high (0.2, 0.3 and 0.4 m). *K*_*tads*_ was set to 1,000 individuals. For each unique combination of parameters (Table S2), we performed 100 replicate model simulations.

### 2.9 Mixed within‐population susceptibility

We next manipulated the variance and distribution of *GSM* values within a population. Because the distribution of *GSM* values in natural populations remains uncharacterized, we tested three different *GSM* distributions within a population: uniform, normal and log‐normal. For the log‐normal scenarios, the distribution was skewed towards high *GSM* values simulating scenarios where (i) novel mutations have produced a small number of individuals with low susceptibility, (ii) individuals with low susceptibility have recently emigrated from a neighbouring population or (iii) the population previously experienced conditions where there was a cost associated with low susceptibility. For all three distributions, we fixed the mean *GSM* value to 0.5 and tested six different variance scenarios creating populations that ranged from having individuals that encompassed all of the parameter space (*GSM* = 0–1) to populations with almost no variation (*GSM *= 0.49–0.51). For all simulations, *K*_*adults*_ was 200, *K*_*tads*_ was 1,000, and transmission was moderate (0.3 m; Table S2). We performed 100 replicates for each combination of parameters.

### 2.10 Exploring parameter space

We next modelled 7,098 combinations of parameter values to determine the effect of each variable in contributing to population outcome. We varied transmission from low to very high (13 levels), *K*_*adults*_ from 50 to 1,200 individuals (seven levels) and *K*_*tads*_ from 50 to 5,000 individuals (six levels). Additionally, we varied the number of unique *GSM* values in the population from 0 to 30 (genetic diversity; 13 levels). To identify the *GSM* values to use in each population, we randomly selected values from a uniform distribution spanning 0–1 with values every 0.001. GSM values were independently selected for each simulation such that each population had a unique composition of initial *GSM* values, even if they contained the same number of *GSM* values. Each unique parameter set (Table S2) was replicated 80 times.

To illustrate the main effects, we plotted each variable against the population outcome without holding the other variables constant. This procedure, which can be considered a type of sensitivity analysis, reflects a summary of each variable across a large exploration of the total parameter space. We did not perform any significance tests on our data (e.g., calculate *p*‐values) as such analyses can be biased by the large number of replicates (White, Rassweiler, Samhouri, Stier, & White, 2014).

## 3 RESULTS

### 3.1 Fixed within‐population susceptibility

**Figure 2:**
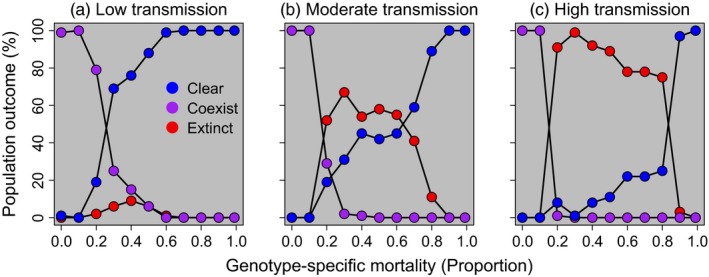
Population outcomes when genotype‐specific mortality (*GSM*) is fixed within a population (i.e., no host evolution). For each value of *GSM,* we performed 100 replicate simulations and recorded the population outcome. Blue points represent the per cent of host populations that cleared infection, purple points the per cent of host populations that coexisted with *Bd*, and red points the per cent of host populations that went extinct. Panels (a), (b) and (c) represent low (0.2 m), moderate (0.3 m) and high (0.4 m) transmission, respectively. All populations had a carrying capacity of 200 adults and 1,000 tadpoles. Notice that intermediate levels of genotype‐specific mortality and higher transmission resulted in a greater proportion of extinctions

The highest proportion of populations that cleared infection occurred at high levels of *GSM*, coexistence was highest at low levels of *GSM*, and extinction was greatest at intermediate levels of *GSM* (Figure 2). Additionally, there were substantially more population extinctions as *Bd* transmission increased; higher transmission increased both the frequency of population extinction at a given *GSM* value and the range of *GSM* values at which extinctions occurred (cf. Figure 2a‐c). Similar relationships between *GSM* and population outcome were found when *K*_*adults*_ was set to 100 or 300 (Fig. S3) or when we increased the number of infected individuals initially introduced into the population (Fig. S4).

**Figure 3:**
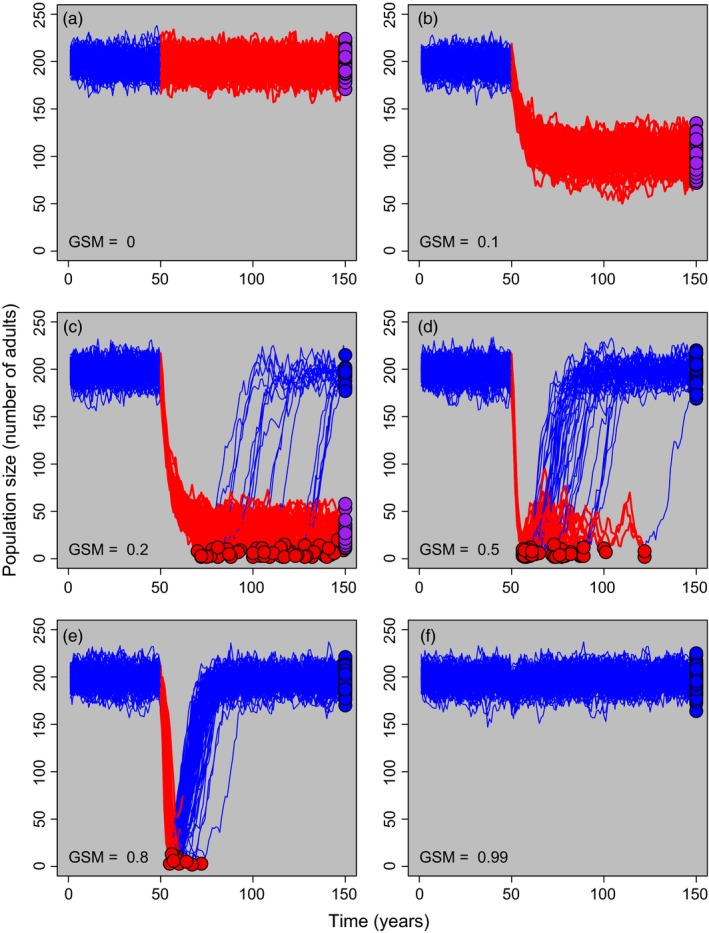
Host population size (number of adults) through time for six different values of genotype‐specific mortality (*GSM*; indicated in the bottom left of each panel) when all individuals within the population have the same *GSM*. Each line represents a single model run. *Bd* was introduced into the population in year 50. Blue lines represent uninfected host populations while red lines represent populations with at least one infected individual. Dots represent the population outcome where red points (jittered) show host populations that went extinct, blue points show host populations that cleared infection, and purple points illustrate host populations that coexisted with infection. Model parameters were set to default values (*K*_*adults*_ = 200, *K*_*tads *_= 1,000 and moderate transmission as in Figure 2b)

These population outcomes were observed because at very low levels of *GSM*, there was little to no *Bd*‐related mortality and adult population sizes remained close to *K*_*adults*_ (Figure 3, Fig. S5, S6), leading to high rates of coexistence. At intermediate *GSM* levels, enough individuals survived each year to maintain the pathogen in the population, but mortality was high enough that adult population sizes were greatly reduced, increasing the chances of extinction. When *GSM* was near one, many infected hosts died before transmitting *Bd*, leading to the population clearing infection so quickly that at most, a small, temporary reduction in adult population size was observed (e.g., Figure 3f). In all scenarios, populations that cleared *Bd* eventually returned to a population abundance near *K*_*adults*_ (Figure 3).

**Figure 4:**
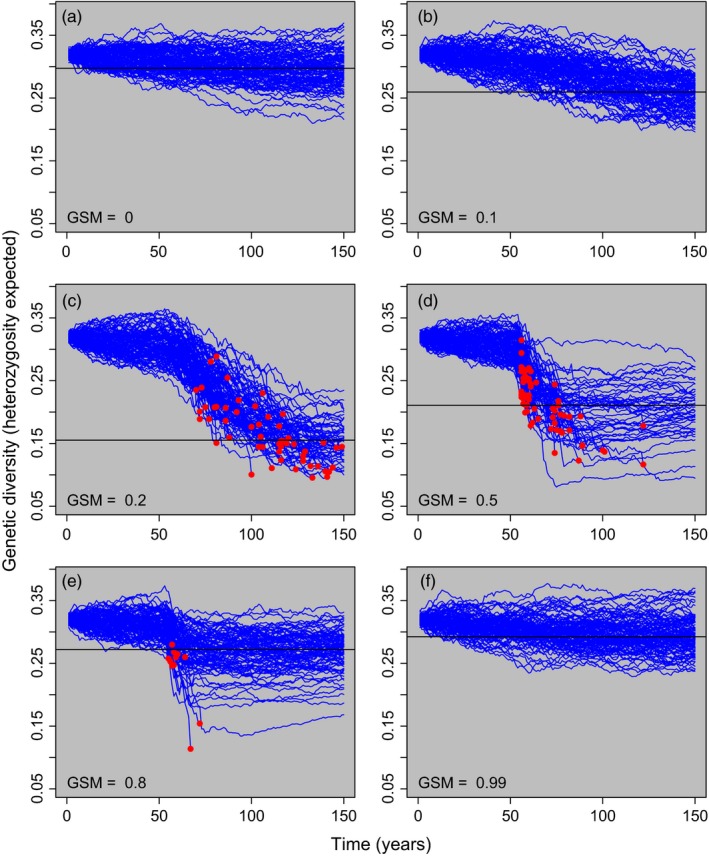
Genetic diversity through time for six different values of genotype‐specific mortality (*GSM*; indicated in the bottom left of each panel). Each blue line represents a single model run. *Bd* was introduced into the population in year 50. Genetic diversity was measured as expected heterozygosity at the 100 neutral SNP loci. Red points highlight host populations that went extinct. The solid horizontal line in each panel indicates the median heterozygosity of populations that persisted for all 150 years. The populations illustrated in this figure are the same as those illustrated in Figure 3. Notice that the loss of genetic diversity is greatest in populations with intermediate *GSM* values

The presence of *Bd* also caused the greatest reduction in neutral genetic diversity at intermediate levels of *GSM* (Figure 4, Fig. S7). For example, a *GSM* value of 0.2 or 0.5 resulted in an average 49.5% (*SD *= 12.6) or 34.8% (*SD *= 13.2) reduction in the expected heterozygosity in the presence of *Bd*, respectively (Figure 4c,d). Low or high *GSM* values resulted in only a modest reduction in heterozygosity expected or the proportion of polymorphic loci (Figure 4, Fig. S7). Over the short timescales illustrated here, mutation is unlikely to result in recovery of neutral genetic diversity. However, gene flow from neighbouring populations could mitigate some of the losses in genetic diversity we observed.

### 3.2 Mixed within‐population susceptibility

**Figure 5:**
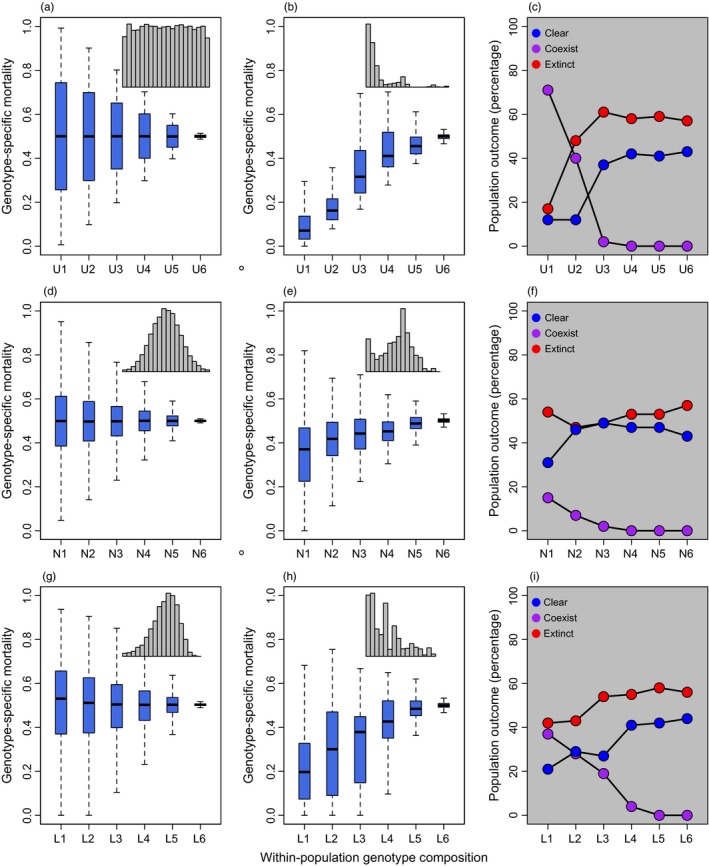
Response of host populations to *Bd* depending upon their within‐population composition of host susceptibility (*GSM*; genotype‐specific mortality). We explored three possible distributions labelled on the *x*‐axis with a “U,” “N” or “L,” to indicate a uniform (a‐c), normal (d‐f) or log‐normal (g‐i) distribution, respectively. The first column (a,d,g) represents the average distribution of *GSM* within populations prior to the introduction of *Bd* (year 49; inset depicts a representative histogram of one population). For each distribution, six different within‐population variances in *GSM* were tested. The second column (b,e,h) illustrates the average distribution of *GSM* 100 years after the introduction of *Bd* or immediately before host population extinction. The third column (c,f,i) illustrates the population outcomes for each population distribution. Host populations with a wider range of susceptibility values can respond to selection and have a much greater rate of coexistence than populations that do not have sufficient adaptive genetic variation to respond to selection

Host populations with wider range in *GSM* values experienced a greater evolutionary response, leading to a greater proportion of host populations coexisting with *Bd* (Figure 5). The uniform distribution for *GSM* resulted in the greatest evolutionary response (Figure 5a,b), followed by the log‐normal distribution (Fig 5g,h) and then the normal distribution (Figure 5d,e). In all cases, the evolution of host populations resulted in a greater proportion of populations coexisting with *Bd* (e.g., populations U1, N1 and L1 in Figure 5). For example, in populations with a uniform *GSM* distribution, there was a 71‐fold increase in the number of populations that coexisted with *Bd* as variation in host susceptibility increased (compare populations U1 to U6). A limited evolutionary response (e.g., populations U6, N6, L6 in Figure 5) led to a higher proportion of populations clearing infection, but also a higher proportion of populations going extinct. Note that populations U6, N6 and L6, which had little initial variation in *GSM* values, show similar population outcomes to each other and to those with a fixed *GSM* value of 0.5 in Figure 2b.

### 3.3 Exploring parameter space

**Figure 6:**
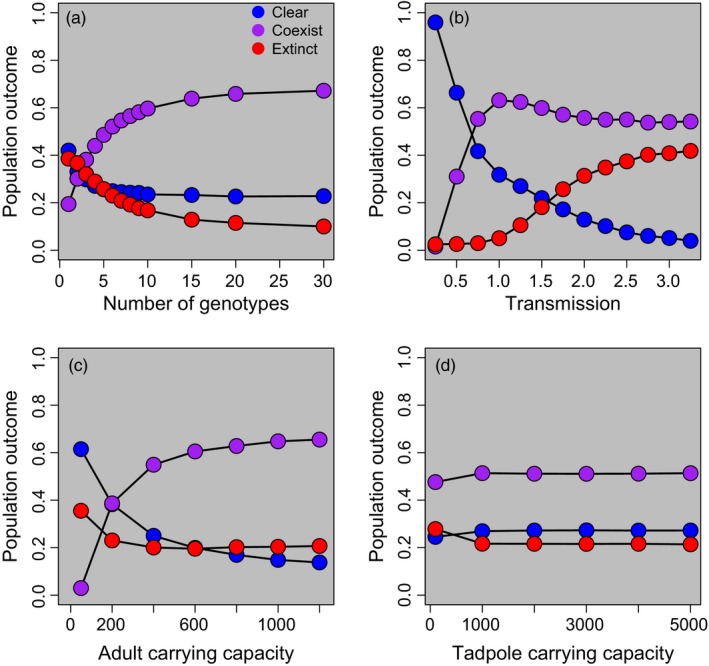
Population outcome across all 7,098 parameter combinations (Table S2). To illustrate the main effects, we plotted each parameter of interest against the proportion of population outcomes while not holding the other variables constant. Results are shown for (a). the number of host genotypes, selected randomly from a uniform distribution of *GSM* values spanning 0–1, (b). transmission, in maximum metres of distance that a zoospore can travel from infected to uninfected individuals (in a 100 × 100 m environment), (c). adult carrying capacity and (d). tadpole carrying capacity. Adult carrying capacity, transmission and the number of genotypes have noticeable effects on population outcome, while tadpole carrying capacity has little to no effect

Across all scenarios, increasing genetic diversity (number of host genotypes) in the population increased rates of coexistence with *Bd* and decreased rates of extinction and clearance (Figure 6a). Changes in genetic diversity had the largest effect in the range of 1–10 genotypes (Figure 6a). Doubling the number of genotypes within the 0–10 range led to a 12.5% increase in the number of coexisting populations and a 7.3% and 5.3% decrease in the number of populations that went extinct or cleared infection, respectively. For the populations that coexisted with *Bd,* we found that higher initial genetic diversity led to a smaller reduction in adult abundance (Fig. S8).

With greater transmission, the proportion of populations that cleared *Bd* decreased rapidly and the proportion of extinctions increased in a sigmoidal fashion (Figure 6b). Rates of coexistence peaked at a transmission value of 1.0 m (i.e., when a *Bd* zoospore could travel up to 1.0 metres to infect a new host). At very low levels of transmission, the pathogen was unable to persist. As transmission increased slightly (e.g., from 0.25 to 1.0 m), *Bd* was able to persist in populations, but rates of infection were low enough that extinction was uncommon, allowing coexistence to occur. However, as transmission continued to increase (over 1.0 m), many individuals became infected, increasing rates of extinction, reducing rates of clearance and slightly reducing rates of coexistence.

*K*_*adults*_ had a much larger effect on population outcome than *K*_*tads*_. Doubling *K*_*adults*_ led to a 14.1% increase in the number of populations coexisting with *Bd*, and a 2.6% and 9.7% decrease in the number experiencing extinction and clearance, respectively (Figure 6c). *K*_*tads*_ had relatively little effect on population outcome (Figure 6d); for each additional tadpole, there was a 5.1 × 10^−6^ and 4.1 × 10^−6^ increase in coexistence and clearance, respectively, and a 9.2 × 10^−6^ decrease in the proportion of extinctions.

## 4 DISCUSSION

A rapid evolutionary response to selection is one mechanism by which host populations can persist after the introduction of a novel pathogen. Our analyses demonstrate that increased variation in amphibian host susceptibility allowed populations to respond faster and to a greater extent to *Bd‐*induced mortality (Figures 5 and 6a). This evolutionary response greatly decreased rates of host extinction and increased rates of host–pathogen coexistence. However, increased coexistence came at a cost of reduced rates of *Bd* clearance, reduced adult population sizes and reduced neutral genetic diversity. We also found that adult carrying capacity and rates of transmission can have large effects on host population outcome, while tadpole population size had a relatively small impact (Figure 6). Our analyses also illustrate the importance of characterizing the distribution of host susceptibility (*GSM)* values within a population; the variance, mean and shape of *GSM* distributions within an amphibian population will directly influence the chances of evolutionary rescue after the introduction of a pathogen (Figure 5).

Given the importance of host susceptibility (*GSM*) in our study, it is imperative that we gather more empirical estimates for this parameter. Susceptibility is a phenotypic trait which is determined by both genetic and environmental contributions. Thus, the first step for comparing *GSM* across populations is to develop a standardized assay to measure this phenotype, although disentangling the genetic and environmental contributions to *GSM* is challenging in systems without genetic clones. While quantitative genetic breeding designs may circumvent some of these challenges, identifying panels of genetic markers that correlate with susceptibility may be a more practical approach (Bataille et al., 2015; Hirschhorn & Daly, 2005; Savage & Zamudio, 2016). With enough individuals, this procedure (or conceptually similar alternatives) will allow for the characterization of the within‐population variation in host susceptibility. Regardless of the chosen approach, there remains a need to empirically quantify the variation in susceptibility within and among amphibian populations and species in order to predict which populations will be able to respond to *Bd*.

When we prevented host evolution from occurring in our model (i.e., when all individuals in a population had the same *GSM* value), extinction was highest at intermediate levels of *GSM* (Figure 2). In these scenarios with fixed *GSM* values throughout the population, *GSM* behaves similarly to pathogen virulence, which is also predicted to have the largest effects on populations at intermediate levels (Anderson, 1979, 1982). Furthermore, this result echoes previous studies of evolutionary rescue, where populations experiencing intermediate levels of disturbance or stress are most likely to benefit from evolutionary rescue (Bell & Gonzalez, 2011). Thus, if there is no (or limited) genetic variation for susceptibility within a host population (e.g., in small host populations), then populations with very high or low levels of susceptibility will be the least likely to go extinct.

Because we focused on evolution of the host, none of our model simulations allowed for evolution of the pathogen. While there is some evidence that pathogen evolution could occur in this system (Piovia‐Scott et al., 2015; Refsnider, Poorten, Langhammer, Burrowes, & Rosenblum, 2015), most amphibian communities contain single or very few strains of *Bd,* such that evolution of the pathogen is expected to be limited (e.g., Jenkinson et al., 2016; Morgan et al., 2007). However, if *Bd* can evolve reduced virulence in a declining host population, we would expect rates of coexistence to increase.

Clearing infection did not require host evolution and, in fact, rapid host evolution promoted coexistence and not clearance (Figures 5 and 6a). Although pathogen clearance from a host population occurred in many of our simulations, clearance of *Bd* has only been documented in the field after substantial intervention (Bosch et al., 2015). Why is clearance so rare in natural populations? Even if populations can theoretically clear infection, the pathogen may persist if it can survive or reproduce outside of the focal host population (e.g., a reservoir host or environmental refuge), which was not present in our model. When this occurs, the pathogen can persist even if the focal host population reaches extremely low densities. An alternative explanation is that researchers are underestimating the rates of *Bd* clearance in the wild, which requires long‐term monitoring for successful detection. For example, some populations in our study did not clear infection until over 50 years after *Bd* introduction (Figure 3c,d). Thus, our results highlight the need for long‐term studies to identify reservoirs of *Bd* and to understand why *Bd* clearance has been so rarely documented in natural populations.

Many of the simulations that led to populations clearing *Bd* also led to reduced neutral genetic diversity. Reduced neutral genetic diversity can affect long‐term population persistence by reducing the ability of populations to respond to future perturbations (e.g., introduction of a different pathogen strain or species). Because neutral genetic diversity and analogous measures (e.g., effective population size) are often used as a proxy for future adaptive potential (Waples, 1990; Reed & Frankham, 2003; but see Mittell, Nakagawa, & Hadfield, 2015), these measures may be useful as a coarse overview of the number of adaptive genotypes present within a population. Additionally, if the introduction of *Bd* is documented within a population, a rapid reduction in genetic diversity may suggest high rates of *Bd*‐associated mortality.

In addition to variation in host susceptibility, transmission was an important factor in our model simulations, in part because our range of transmission values was not bounded above zero. In contrast, many of our other parameters were constrained by definition (e.g*., GSM* can only have values from 0–1) or design (e.g., we chose *K*_*adults*_ values that represent natural population sizes: Table S1). In natural systems, variation in transmission could occur due to environmental conditions or *Bd* strain. Our results show that if *Bd* transmission is low, the chances of host extinction are also low, regardless of the mean and distribution of susceptibilities (Figures 2 and 6b). Additionally, changing the transmission function of our model to include environmental transmission could lead to different results for host evolution and population outcome. In general, allowing for survival of a pathogen in the environment is likely to increase rates of extinction and reduce the chances of pathogen clearance, because transmission can remain high even if host density becomes extremely low. In the *Bd* system, a previous study using a mathematical model found that the longer *Bd* can survive in water, the greater the chances of host extinction (Mitchell, Churcher, Garner, & Fisher, 2008). Furthermore, pathogens that can survive in the environment for long periods of time are expected to evolve higher virulence because host survival has a weaker effect on virulence evolution (reviewed in Cressler, Mcleod, Rozins, Hoogen, & Day, 2016). Thus, identifying ways in which transmission can be reduced in natural populations and the conditions that prevent *Bd* from surviving in the environment could have large effects on the persistence of amphibian populations.

Managing amphibian populations to have large adult population sizes and high genetic diversity may also help reduce the chances of population extinction after the introduction of *Bd*. These are the same conditions that are predicted to increase the chances of evolutionary rescue in previous studies (reviewed in Carlson et al., 2014). In our model, rates of extinction were lowest when populations consisted of a wider range of susceptibility values (Figures 5 and 6a), although these populations coexisted with *Bd* and therefore maintained population sizes below carrying capacity (Figure 3). We also found that increased genetic diversity benefited populations even when the population outcome was the same; populations with greater numbers of host genotypes were able to maintain larger population sizes when coexisting with *Bd* (Fig. S8). Thus, we recommend implementing policies to maintain genetic diversity of threatened populations to reduce their extinction risk from *Bd*. However, managing populations for potential invasion by a novel pathogen can result in conflicting trade‐offs; small populations can be less likely to be colonized by an invasive pathogen than large populations due to the smaller number of potential hosts and reduced capacity for transmission (Burdon, Ericson, & Muller, 1995; Stapp, Antolin, & Ball, 2004). Additionally, higher gene flow into a population will generally increase genetic diversity, but also increased risk of pathogen introduction. Thus, management policies must consider trade‐offs between the risk of *Bd* introduction versus the risk of extinction once *Bd* has already arrived in a population. In the absence of any knowledge regarding the within‐population variation in host susceptibility, we recommend prioritizing conservation of areas with large adult population sizes, limited immigration of potentially infected individuals and high genetic diversity.

While our model was designed to mimic conditions in amphibian populations exposed to *Bd*, similar patterns of host evolution and population outcome are likely in other systems. For example, our model is relevant for infection dynamics in hosts with complex life cycles, particularly if one life stage experiences higher mortality from infection than others (e.g., pathogens that primarily infect seeds; Kremer, 1993; or cause high mortality in adults; McLaughlin & Myers, 1970). Additionally, because our model incorporates two separate environments (aquatic and terrestrial), it can inform host systems that include migration and dispersal, particularly where the risk of transmission is higher in one habitat than the other (e.g., fish with a pelagic larval stage; Poulin et al., 2012; reindeer with postcalving migrations; Folstad, Nilssen, Halvorsen, & Andersen, 1991). However, it is important to note that in our model, hosts varied in their response to infection (i.e*.,* pathogen‐induced mortality; *GSM*), but not in their likelihood of becoming infected. In systems where host defences primarily involve reducing the chances that an individual becomes infected, evolutionary rescue may lead to an increase in clearance and not coexistence. Thus, we predict that many of the qualitative results we found in this study would also be found in other systems, but the commonality of our findings to systems that lack variation in pathogen‐induced mortality or that have different modes of pathogen transmission (e.g., a pathogen that can survive long periods of time in the environment) requires further validation. Because individual‐based models are able to incorporate a multitude of different host, pathogen and environmental scenarios, they continue to be a powerful tool for the study and management of infectious disease in systems with a wide range of characteristics.

### 4.1 Conclusion

In summary, our analyses demonstrate that possible within‐population variation in the susceptibility of amphibian hosts to *Bd* could play a large role in evolutionary rescue. Rapid evolutionary responses have been increasingly documented in diverse taxa such as plants (Franks, Sim, & Weis, 2007), fishes (Christie et al., 2016; Lescak et al., 2015), lizards (Campbell‐Staton et al., 2017) and birds (Grant & Grant, 2006), suggesting that many host species have the potential to rapidly respond to pathogen‐mediated selection. However, here we show that in host–pathogen systems, evolutionary rescue can lead to increased rates of coexistence and decreased rates of extinction. Such evolutionary rescue, however, is not without trade‐offs; decreased population sizes and lower rates of clearing infection are the costs associated with this process. Nevertheless, for a virulent pathogen that has extirpated large numbers of populations, the drawbacks associated with coexistence greatly outweigh increased risk of extinction. Thus, the successful conservation and management of amphibians in the wake of a *Bd* epidemic should focus on maintaining genetic diversity, bolstering adult population sizes, and minimizing disease transmission to prevent future extinctions and extirpations of threatened and vulnerable amphibians.

## DATA ACCESSIBILITY

The data sets produced from this work and code for the model are available from the Dryad Digital Repository: https://doi.org/10.5061/dryad.pc054.

## References

[eva12568-bib-0001] Aguirre, A. A. , & Tabor, G. M. (2008). Global factors driving emerging infectious diseases. Annals of the New York Academy of Sciences, 1149, 1–3. https://doi.org/10.1196/nyas.2008.1149.issue-1 19120161 10.1196/annals.1428.052

[eva12568-bib-0002] Altermatt, F. , & Ebert, D. (2008). Genetic diversity of *Daphnia magna* populations enhances resistance to parasites. Ecology Letters, 11, 918–928. https://doi.org/10.1111/ele.2008.11.issue-9 18479453 10.1111/j.1461-0248.2008.01203.x

[eva12568-bib-0003] Altizer, S. , Harvell, D. , & Friedle, E. (2003). Rapid evolutionary dynamics and disease threats to biodiversity. Trends in Ecology & Evolution, 18, 589–596. https://doi.org/10.1016/j.tree.2003.08.013

[eva12568-bib-0004] Anderson, R. M. (1979). Parasite pathogenicity and the depression of host population equilibria. Nature, 279, 150–152. https://doi.org/10.1038/279150a0

[eva12568-bib-0005] Anderson, R. M. (1982). Theoretical basis for the use of pathogens as biological control agents of pest species. Parasitology, 84, 3–33. https://doi.org/10.1017/S0031182000053592

[eva12568-bib-0006] Anderson, J. D. , Hassinge, D. D. , & Dalrymple, G. H. (1971). Natural mortality of eggs and larvae of *Ambystoma t. tigrinum* . Ecology, 52, 1107–1112. https://doi.org/10.2307/1933820

[eva12568-bib-0007] Bancroft, B. A. , Han, B. A. , Searle, C. L. , Biga, L. M. , Olson, D. H. , Kats, L. B. , … Blaustein, A. R. (2011). Species‐level correlates of susceptibility to the pathogenic amphibian fungus *Batrachochytrium dendrobatidis* in the United States. Biodiversity and Conservation, 20, 1911–1920. https://doi.org/10.1007/s10531-011-0066-4

[eva12568-bib-0008] Bataille, A. , Cashins, S. D. , Grogan, L. , Skerratt, L. F. , Hunter, D. , McFadden, M. , … Waldman, B. (2015). Susceptibility of amphibians to chytridiomycosis is associated with MHC class II conformation. Proceedings. Biological Science, 282, 20143127 https://doi.org/10.1098/rspb.2014.3127 10.1098/rspb.2014.3127 PMC4389617 25808889

[eva12568-bib-0009] Bell, G. (2013). Evolutionary rescue and the limits of adaptation. Philosophical Transactions of the Royal Society of London. Series B, Biological sciences, 368, 20120080 https://doi.org/10.1098/rstb.2012.0080 23209162 10.1098/rstb.2012.0080 PMC3538447

[eva12568-bib-0010] Bell, G. , & Gonzalez, A. (2011). Adaptation and evolutionary rescue in metapopulations experiencing environmental deterioration. Science, 322, 1327–1330. https://doi.org/10.1126/science.1203105 10.1126/science.1203105 21659606

[eva12568-bib-0011] Bonte, D. , Hovestadt, T. , & Poetke, H. J. (2009). Sex‐specific dispersal and evolutionary rescue in metapopulations infected by male killing endosymbionts. BMC Evolutionary Biology, 9, 16 https://doi.org/10.1186/1471-2148-9-16 19149895 10.1186/1471-2148-9-16 PMC2633281

[eva12568-bib-0012] Bosch, J. , Sanchez‐Tome, E. , Fernandez‐Loras, A. , Oliver, J. A. , Fisher, M. C. , & Garner, W. J. (2015). Successful elimination of a lethal wildlife infectious disease in nature. Biology Letters, 11, 20150874 https://doi.org/10.1098/rsbl.2015.0874 26582843 10.1098/rsbl.2015.0874 PMC4685552

[eva12568-bib-0013] Boyle, D. G. , Hyatt, A. D. , Daszak, P. , Berger, L. , Longcore, J. E. , Porter, D. , … Olsen, V. (2003). Cryo‐archiving of *Batrachochytrium dendrobatidis* and other chytridiomycetes. Diseases of Aquatic Organisms, 56, 59–64. https://doi.org/10.3354/dao056059 14524502 10.3354/dao056059

[eva12568-bib-0014] Briggs, C. J. , Knapp, R. A. , & Vredenburg, V. T. (2010). Enzootic and epizootic dynamics of the chytrid fungal pathogen of amphibians. Proceedings of the National Academy of the Sciences USA, 107, 9695–9700. https://doi.org/10.1073/pnas.0912886107 10.1073/pnas.0912886107 PMC2906864 20457916

[eva12568-bib-0015] Briggs, C. J. , Vredenburg, V. T. , Knapp, R. A. , & Rachowicz, L. J. (2005). Investigating the population‐level effects of chytridiomycosis: An emerging infectious disease of amphibians. Ecology, 86, 3149–3159. https://doi.org/10.1890/04-1428

[eva12568-bib-0016] Burdon, J. J. , & Chilvers, G. A. (1982). Host density as a factor in plant disease ecology. Annual Review of Phytopathology, 20, 143–166. https://doi.org/10.1146/annurev.py.20.090182.001043

[eva12568-bib-0017] Burdon, J. J. , Ericson, L. , & Muller, W. J. (1995). Temporal and spatial changes in a metapopulation of the rust pathogen *Triphragmium ulmariae* and its host, *Filipendula ulmaria* . Journal of Ecology, 83, 979–989. https://doi.org/10.2307/2261179

[eva12568-bib-0018] Calef, G. W. (1973). Natural mortality of tadpoles in a population of *Rana aurora* . Ecology, 54, 741–758. https://doi.org/10.2307/1935670

[eva12568-bib-0019] Campbell‐Staton, S. C. , Cheviron, Z. A. , Rochette, N. , Catchen, J. , Losos, J. B. , & Edwards, S. V. (2017). Winter storms drive rapid phenotypic, regulatory, and genomic shifts in the green anole lizard. Science, 357, 495–498. https://doi.org/10.1126/science.aam5512 28774927 10.1126/science.aam5512

[eva12568-bib-0020] Carlson, S. M. , Cunningham, C. J. , & Westley, P. A. H. (2014). Evolutionary rescue in a changing world. Trends in Ecology and Evolution, 29, 521–530. https://doi.org/10.1016/j.tree.2014.06.005 25038023 10.1016/j.tree.2014.06.005

[eva12568-bib-0021] Christie, M. R. , Marine, M. Ll. , Fox, S. E. , French, R. A. , & Blouin, M. S. (2016). A single generation of domestication heritably alters the expression of hundreds of genes. Nature Communications, 7, 10676 https://doi.org/10.1038/ncomms10676 10.1038/ncomms10676 PMC4757788 26883375

[eva12568-bib-0022] Christie, M. R. , Marine, M. L. , French, R. A. , Waples, R. S. , & Blouin, M. S. (2012). Effective size of a wild salmonid population is greatly reduced by hatchery supplementation. Heredity, 109, 254–260. https://doi.org/10.1038/hdy.2012.39 22805657 10.1038/hdy.2012.39 PMC3464026

[eva12568-bib-0023] Converse, S. J. , Bailey, L. L. , Mosher, B. A. , Funk, W. C. , Gerber, B. D. , & Muths, E. (2016). A model to inform management actions as a response to chytridiomycosis‐associated decline. EcoHealth, https://doi.org/10.1007/s10393-016-1117-9 10.1007/s10393-016-1117-9 27056609

[eva12568-bib-0024] Cressler, C. E. , Mcleod, D. V. , Rozins, C. , Hoogen, J. V. D. , & Day, T. (2016). The adaptive evolution of virulence: A review of theoretical predictions and empirical tests. Parasitology, 143, 915–930. https://doi.org/10.1017/S003118201500092X 26302775 10.1017/S003118201500092X PMC4873896

[eva12568-bib-0025] Daszak, P. (2000). Emerging infectious diseases of wildlife ‐ Threats to biodiversity and human health. Science, 287, 1756–1756. 10.1126/science.287.5452.443 10642539

[eva12568-bib-0026] Daszak, P. , Scott, D. E. , Kilpatrick, A. M. , Faggioni, C. , Gibbons, J. W. , & Porter, D. (2005). Amphibian population declines at savannah river site are linked to climate, not chytridiomycosis. Ecology, 86, 3232–3237. https://doi.org/10.1890/05-0598

[eva12568-bib-0027] Daszak, P. , Strieby, A. , Cunningham, A. A. , Longcore, J. E. , Brown, C. C. , & Porter, D. (2004). Experimental evidence that the bullfrog (*Rana catesbeiana*) is a potential carrier of chytridiomycosis, an emerging fungal disease of amphibians. Herpetological Journal, 14, 201–207.

[eva12568-bib-0028] DeAngelis, D. L. , & Grimm, V. (2014). Individual‐based models in ecology after four decades. F1000prime Reports, 6, 39 https://doi.org/10.12703/P6-39 24991416 10.12703/P6-39 PMC4047944

[eva12568-bib-0029] Drawert, B. , Griesemer, M. , Petzold, L. R. , & Briggs, C. J. (2017). Using stochastic epidemiological models to evaluate conservation strategies for endangered amphibians. Journal of the Royal Society Interface, 14, 20170480 https://doi.org/10.1098/rsif.2017.0480 10.1098/rsif.2017.0480 PMC5582134 28855388

[eva12568-bib-0030] Duffy, M. A. , Ochs, J. H. , Penczykowski, R. M. , Civitello, D. J. , Klausmeier, C. A. , & Hall, S. R. (2012). Ecological context influences epidemic size and parasite‐driven evolution. Science, 335, 1636–1638. https://doi.org/10.1126/science.1215429 22461614 10.1126/science.1215429

[eva12568-bib-0031] Dunlop, E. S. , Baskett, M. L. , Heino, M. , & Dieckmann, U. (2009). Propensity of marine reserves to reduce the evolutionary effects of fishing in a migratory species. Evolutionary Applications, 2, 371–393. https://doi.org/10.1111/j.1752-4571.2009.00089.x 25567887 10.1111/j.1752-4571.2009.00089.x PMC3352486

[eva12568-bib-0032] Dunlop, E. S. , Heino, M. , & Dieckmann, U. (2009). Eco‐genetic modeling of contemporary life‐history evolution. Ecological Applications, 19, 1815–1834. https://doi.org/10.1890/08-1404.1 19831072 10.1890/08-1404.1

[eva12568-bib-0033] Folstad, I. , Nilssen, A. C. , Halvorsen, O. , & Andersen, J. (1991). Parasite avoidance: The cause of post‐calving migrations in *Rangifer*? Canadian Journal of Zoology, 69, 2423–2429. https://doi.org/10.1139/z91-340

[eva12568-bib-0034] Franks, S. J. , Sim, S. , & Weis, A. E. (2007). Rapid evolution of flowering time by an annual plant in response to a climate fluctuation. Proceedings of the National Academy of the Sciences USA, 104, 1278–1282. https://doi.org/10.1073/pnas.0608379104 10.1073/pnas.0608379104 PMC1783115 17220273

[eva12568-bib-0035] Gandon, S. , Hochberg, M. E. , Holt, R. D. , & Day, T. (2012). What limits the evolutionary emergence of pathogens? Philosophical transactions of the Royal Society of London. Series B, Biological sciences, 368, 20120086 https://doi.org/10.1098/rstb.2012.0086 10.1098/rstb.2012.0086 PMC3538453 23209168

[eva12568-bib-0036] Garner, T. W. J. , Walker, S. , Bosch, J. , Leech, S. , Rowcliffe, J. M. , Cunningham, A. A. , & Fisher, M. C. (2009). Life history tradeoffs influence mortality associated with the amphibian pathogen *Batrachochytrium dendrobatidis* . Oikos, 118, 783–791. https://doi.org/10.1111/oik.2009.118.issue-5

[eva12568-bib-0037] Gonzalez, A. , Ronce, O. , Ferriere, R. , & Hochberg, M. E. (2012). Evolutionary rescue: An emerging focus at the intersection between ecology and evolution. Philosophical transactions of the Royal Society of London. Series B, Biological sciences, 368, 20120404 https://doi.org/10.1098/rstb.2012.0404 10.1098/rstb.2012.0404 PMC3538460 23209175

[eva12568-bib-0038] Grant, P. R. , & Grant, B. R. (2006). Evolution of character displacement in Darwin's Finches. Science, 313, 224 https://doi.org/10.1126/science.1128374 16840700 10.1126/science.1128374

[eva12568-bib-0039] Grogan, L. F. , Phillott, A. D. , Scheele, B. C. , Berger, L. , Cashins, S. D. , Bell, S. C. , … Skerratt, L. F. (2016). Endemicity of chytridiomycosis features pathogen overdispersion. Journal of Animal Ecology, 85, 806–816. https://doi.org/10.1111/1365-2656.12500 26847143 10.1111/1365-2656.12500

[eva12568-bib-0040] Harvell, C. D. , Mitchell, C. E. , Ward, J. R. , Altizer, S. , Dobson, A. P. , Ostfeld, R. S. , Samuel, M. D. (2002). Climate warming and disease risks for terrestrial and marine biota. Science, 296, 2158–2162. https://doi.org/10.1126/science.1063699 12077394 10.1126/science.1063699

[eva12568-bib-0041] Hedrick, P. (2005). Large variance in reproductive success and the N‐e/N ratio. Evolution, 59, 1596–1599. https://doi.org/10.1111/evo.2005.59.issue-7 16153045

[eva12568-bib-0042] Hendry, A. P. (2016). Eco‐evolutionary dynamics. Princeton, N.J., USA: Princeton University Press.

[eva12568-bib-0043] Hirschhorn, J. N. , & Daly, M. J. (2005). Genome‐wide association studies for common diseases and complex traits. Nature Reviews Genetics, 6, 95–108. https://doi.org/10.1038/nrg1521 10.1038/nrg1521 15716906

[eva12568-bib-0044] Hughes, W. O. H. , & Boomsma, J. J. (2004). Genetic diversity and disease resistance in leaf‐cutting ant societies. Evolution, 58, 1251–1260. https://doi.org/10.1111/evo.2004.58.issue-6 15266974 10.1554/03-546

[eva12568-bib-0045] Jenkinson, T. S. , Betancourt Román, C. M. , Lambertini, C. , Valencia‐Aguilar, A. , Rodriguez, D. , Nunes‐de‐Almeida, C. H. L. , … James, T. Y. (2016). Amphibian‐killing chytrid in Brazil comprises both locally endemic and globally expanding populations. Molecular Ecology, 25, 2978–2996. https://doi.org/10.1111/mec.13599 26939017 10.1111/mec.13599

[eva12568-bib-0046] Knapp, R. A. , Fellers, G. M. , Kleeman, P. M. , Miller, D. A. W. , Vredenburg, V. T. , Rosenblum, E. B. , Briggs, C. J. (2016). Large‐scale recovery of an endangered amphibian despite ongoing exposure to multiple stressors. Proceedings of the National Academy of Sciences of the United States of America, 113, 11889–11894. https://doi.org/10.1073/pnas.1600983113 27698128 10.1073/pnas.1600983113 PMC5081604

[eva12568-bib-0047] Kremer, R. J. (1993). Management of weed seed banks with microorganisms. Ecological Applications, 3, 42–52. https://doi.org/10.2307/1941791 27759226 10.2307/1941791

[eva12568-bib-0048] Lescak, E. A. , Bassham, S. L. , Catchen, J. , Gelmond, O. , Sherbick, M. L. , von Hippel, F. A. , & Cresko, W. A. (2015). Evolution of stickleback in 50 years on earthquake‐uplifted islands. Proceedings of the National Academy of Sciences, 112, E7204–E7212. https://doi.org/10.1073/pnas.1512020112 10.1073/pnas.1512020112 PMC4702987 26668399

[eva12568-bib-0049] Lively, C. M. (2010). The effect of host genetic diversity on disease spread. The American Naturalist, 175, E149–E152. https://doi.org/10.1086/652430 10.1086/652430 20388005

[eva12568-bib-0050] McLaughlin, R. E. , & Myers, J. (1970). *Ophryocystis elektroscirrha* sp. n., a neogregarine pathogen of the monarch butterfly *Danus plexippus* (L.) and the Florida queen butterfly *D. gilippus bernice* Cramer. Journal of Eukaryotic Microbiology, 17, 300–305. https://doi.org/10.1111/j.1550-7408.1970.tb02375.x

[eva12568-bib-0051] Mitchell, K. M. , Churcher, T. S. , Garner, T. W. J. , & Fisher, M. C. (2008). Persistence of the emerging pathogen *Batrachochytrium dendrobatidis* outside the amphibian host greatly increases the probability of host extinction. Proceedings of the Royal Society B: Biological Sciences, 275, 329–334. https://doi.org/10.1098/rspb.2007.1356 18048287 10.1098/rspb.2007.1356 PMC2593721

[eva12568-bib-0052] Mittell, E. A. , Nakagawa, S. , & Hadfield, J. D. (2015). Are molecular markers useful predictors of adaptive potential? Ecology Letters, 18, 772–778. https://doi.org/10.1111/ele.2015.18.issue-8 25989024 10.1111/ele.12454

[eva12568-bib-0053] Morgan, J. A. T. , Vredenburg, V. T. , Rachowicz, L. J. , Knapp, R. A. , Stice, M. J. , Tunstall, T. , … Taylor, J. W . (2007). Population genetics of the frog‐killing fungus *Batrachochytrium dendrobatidis* . Proceedings of the National Academy of Sciences of the United States of America, 104, 13845–13850. https://doi.org/10.1073/pnas.0701838104 17693553 10.1073/pnas.0701838104 PMC1945010

[eva12568-bib-0054] Nath, M. , Woolliams, J. A. , & Bishop, S. C. (2008). Assessment of the dynamics of microparasite infections in genetically homogeneous and heterogeneous populations using a stochastic epidemic model. Journal of Animal Science, 86, 1747–1757. https://doi.org/10.2527/jas.2007-0615 18407996 10.2527/jas.2007-0615

[eva12568-bib-0055] Osmond, M. M. , & Mazancourt, C. (2013). How competition affects evolutionary rescue. Philosophical Transactions of the Royal Society of London. Series B, Biological sciences, 368, 20120085 https://doi.org/10.1098/rstb.2012.0085 23209167 10.1098/rstb.2012.0085 PMC3538452

[eva12568-bib-0056] Palomar, G. , Bosch, J. , & Cano, J. M. (2016). Heritability of *Batrachochytrium dendrobatidis* burden and its genetic correlation with development time in a population of common toad (*Bufo spinosus*). Evolution, 70, 2346–2356. https://doi.org/10.1111/evo.2016.70.issue-10 27480345 10.1111/evo.13029

[eva12568-bib-0057] Pederson, A. B. , & Fenton, A. (2015). The role of antiparasite treatment experiments in assessing the impact of parasites on wildlife. Trends in Parasitology, 31, 200–211. https://doi.org/10.1016/j.pt.2015.02.004 25778845 10.1016/j.pt.2015.02.004

[eva12568-bib-0058] Penczykowski, R. M. , Forde, S. E. , & Duffy, M. A. (2011). Rapid evolution as a possible constraint on emerging infectious diseases. Freshwater Biology, 56, 689–704. https://doi.org/10.1111/fwb.2011.56.issue-4

[eva12568-bib-0059] Phillott, A. D. , Grogan, L. F. , Cashins, S. , McDonald, K. R. , Berger, L. , & Skerratt, L. F. (2013). Chytridiomycosis and seasonal mortality of tropical stream‐associated frogs 15 years after introduction of *Batrachochytrium dendrobatidis* . Conservation Biology, 27, 1058–1068. https://doi.org/10.1111/cobi.12073 23678872 10.1111/cobi.12073

[eva12568-bib-0060] Piotrowski, J. S. , Annis, S. L. , & Longcore, J. E. (2004). Physiology of *Batrachochytrium dendrobatidis*, a chytrid pathogen of amphibians. Mycologia, 96, 9–15. https://doi.org/10.1080/15572536.2005.11832990 21148822

[eva12568-bib-0061] Piovia‐Scott, J. , Pope, K. , Joy Worth, S. , Rosenblum, E. B. , Poorten, T. , Refsnider, J. , … Foley, J. (2015). Correlates of virulence in a frog‐killing fungal pathogen: Evidence from a California amphibian decline. ISME Journal, 9, 1570–1578. https://doi.org/10.1038/ismej.2014.241 25514536 10.1038/ismej.2014.241 PMC4478697

[eva12568-bib-0062] Poulin, R. , Closs, G. P. , Lill, A. W. , Hicks, A. S. , Herrmann, K. K. , & Kelly, D. W. (2012). Migration as an escape from parasitism in New Zealand galaxiid fishes. Oecologia, 169, 955–963. https://doi.org/10.1007/s00442-012-2251-x 22271201 10.1007/s00442-012-2251-x

[eva12568-bib-0063] R Core Team (2016). R: A language and environment for statistical computing. Vienna, Austria: R Foundation for Statistical Computing.

[eva12568-bib-0064] Rachowicz, L. J. , & Briggs, C. J. (2007). Quantifying the disease transmission function: Effects of density on *Batrachochytrium dendrobatidis* transmission in the mountain yellow‐legged frog *Rana muscosa* . Journal of Animal Ecology, 76, 711–721. https://doi.org/10.1111/jae.2007.76.issue-4 17584377 10.1111/j.1365-2656.2007.01256.x

[eva12568-bib-0065] Reed, D. H. , & Frankham, R. (2003). Correlation between fitness and genetic diversity. Conservation Biology, 17, 230–237. https://doi.org/10.1046/j.1523-1739.2003.01236.x

[eva12568-bib-0066] Reeder, N. M. M. , Pessier, A. P. , & Vredenburg, V. T. (2012). A reservoir species for the emerging amphibian pathogen *Batrachochytrium dendrobatidis* thrives in a landscape decimated by disease. PLoS One, 7, e33567 https://doi.org/10.1371/journal.pone.0033567 22428071 10.1371/journal.pone.0033567 PMC3299797

[eva12568-bib-0067] Refsnider, J. M. , Poorten, T. J. , Langhammer, P. F. , Burrowes, P. A. , & Rosenblum, E. B. (2015). Genomic correlates of virulence attenuation in the deadly amphibian chytrid fungus, *Batrachochytrium dendrobatidis* . G3 (Bethesda, Md.)., 5(11), 2291–2298. https://doi.org/10.1534/g3.115.021808 10.1534/g3.115.021808 PMC4632049 26333840

[eva12568-bib-0068] Savage, A. E. , & Zamudio, K. R. (2011). MHC genotypes associate with resistance to a frog‐killing fungus. Proceedings of the National Academy of Sciences of the United States of America, 108, 16705–16710. https://doi.org/10.1073/pnas.1106893108 21949385 10.1073/pnas.1106893108 PMC3189034

[eva12568-bib-0069] Savage, A. E. , & Zamudio, K. R. (2016). Adaptive tolerance to a pathogenic fungus drives major histocompatibility complex evolution in natural amphibian populations. Proceedings. Biological Sciences, 283, 20153115 https://doi.org/10.1098/rspb.2015.3115 27009220 10.1098/rspb.2015.3115 PMC4822461

[eva12568-bib-0070] Searle, C. L. , Biga, L. M. , Spatafora, J. W. , & Blaustein, A. R. (2011). A dilution effect in the emerging amphibian pathogen *Batrachochytrium dendrobatidis* . Proceedings of the National Academy of Sciences of the United States of America, 108, 16322–16326. https://doi.org/10.1073/pnas.1108490108 21930900 10.1073/pnas.1108490108 PMC3182747

[eva12568-bib-0071] Searle, C. L. , Gervasi, S. S. , Hua, J. , Hammond, J. I. , Relyea, R. A. , Olson, D. H. , Blaustein, A. R. (2011). Differential host susceptibility to *Batrachochytrium dendrobatidis*, an emerging amphibian pathogen. Conservation Biology, 25, 965–974. https://doi.org/10.1111/j.1523-1739.2011.01708.x 21732979 10.1111/j.1523-1739.2011.01708.x

[eva12568-bib-0072] Skerratt, L. F. , Berger, L. , Speare, R. , Cashins, S. , McDonald, K. R. , Phillott, A. D. , … Kenyon, N. (2007). Spread of chytridiomycosis has caused the rapid global decline and extinction of frogs. EcoHealth, 4, 125–134. https://doi.org/10.1007/s10393-007-0093-5

[eva12568-bib-0073] Smith, K. F. , Sax, D. F. , & Lafferty, K. D. (2006). Evidence for the role of infectious disease in species extinction and endangerment. Conservation Biology, 20, 1349–1357. https://doi.org/10.1111/cbi.2006.20.issue-5 17002752 10.1111/j.1523-1739.2006.00524.x

[eva12568-bib-0074] Springbett, A. J. , MacKenzie, K. , Woolliams, J. A. , & Bishop, S. C. (2003). The contribution of genetic diversity to the spread of infectious diseases in livestock populations. Genetics, 165, 1465–1474. 14668395 10.1093/genetics/165.3.1465 PMC1462849

[eva12568-bib-0075] Stapp, P. , Antolin, M. F. , & Ball, M. (2004). Patterns of extinction in prairie dog metapopulations: Plague outbreaks follow El Nino events. Frontiers in Ecology and the Environment, 2, 235–240. https://doi.org/10.2307/3868263

[eva12568-bib-0076] Stewart, G. S. , Morris, M. R. , Genis, A. B. , Szűcs, M. , Melbourne, B. A. , Tavener, S. J. , & Hufbauer, R. A. (2017). The power of evolutionary rescue is constrained by genetic load. Evolutionary Applications, 10(7), 731–741. https://doi.org/10.1111/eva.12489 28717392 10.1111/eva.12489 PMC5511356

[eva12568-bib-0077] Stockwell, C. A. , Hendry, A. P. , & Kinnison, M. T. (2003). Contemporary evolution meets conservation biology. Trends in Ecology & Evolution, 18, 94–101. https://doi.org/10.1016/S0169-5347(02)00044-7

[eva12568-bib-0078] Venesky, M. D. , Liu, X. , Sauer, E. L. , & Rohr, J. R. (2014). Linking manipulative experiments to field data to test the dilution effect. Journal of Animal Ecology, 83, 557–565. https://doi.org/10.1111/jane.2014.83.issue-3 24289288 10.1111/1365-2656.12159

[eva12568-bib-0079] Voyles, J. , Johnson, L. R. , Briggs, C. J. , Cashins, S. D. , Alford, R. A. , Berger, L. , … Rosenblum, E. B. (2012). Temperature alters reproductive life history patterns in *Batrachochytrium dendrobatidis* a lethal pathogen associated with the global loss of amphibians. Ecology and Evolution, 2, 2241–2249. https://doi.org/10.1002/ece3.2012.2.issue-9 23139882 10.1002/ece3.334 PMC3488674

[eva12568-bib-0080] Waples, R. S. (1990). Conservation genetics of Pacific salmon. II. Effective population size and the rate of loss of genetic variability. Journal of Heredity, 81, 267–276. https://doi.org/10.1093/oxfordjournals.jhered.a110989

[eva12568-bib-0081] Wells, K. D. (1977). The social behaviour of anuran amphibians. Animal Behaviour, 25, 666–693. https://doi.org/10.1016/0003-3472(77)90118-X

[eva12568-bib-0082] Wells, K. D. (2007). The ecology and behavior of amphibians. Chicago, IL: University of Chicago Press https://doi.org/10.7208/chicago/9780226893334.001.0001

[eva12568-bib-0083] White, J. W. , Rassweiler, A. , Samhouri, J. F. , Stier, A. C. , & White, C. (2014). Ecologists should not use statistical significance tests to interpret simulation model results. Oikos, 123, 385–388. https://doi.org/10.1111/more.2014.123.issue-4

[eva12568-bib-0084] Wilber, M. Q. , Langwig, K. E. , Kilpatrick, A. M. , McCallum, H. I. , & Briggs, C. J. (2016). Integral projection models for host–parasite systems with an application to amphibian chytrid fungus. Methods in Ecology and Evolution, 7(10), 1182–1194. https://doi.org/10.1111/2041-210X.12561 28239442 10.1111/2041-210X.12561 PMC5321654

[eva12568-bib-0085] Woodhams, D. C. , Alford, R. A. , Briggs, C. J. , Johnson, M. , & Rollins‐Smith, L. A. (2008). Life‐history trade‐offs influence disease in changing climates: Strategies of an amphibian pathogen. Ecology, 89, 1627–1639. https://doi.org/10.1890/06-1842.1 18589527 10.1890/06-1842.1

[eva12568-bib-0086] Yates, A. , Antia, R. , & Regoes, R. R. (2006). How do pathogen evolution and host heterogeneity interact in disease emergence? Proceeding. Biological Sciences, 273, 3075–3083. https://doi.org/10.1098/rspb.2006.3681 10.1098/rspb.2006.3681 PMC1679899 17015347

[eva12568-bib-0087] Zhu, Y. Y. , Chen, H. R. , Fan, J. H. , Wang, Y. Y. , Li, Y. , Chen, J. B. , … Mundt, C. C . (2000). Genetic diversity and disease control in rice. Nature, 406, 718–722. https://doi.org/10.1038/35021046 10963595 10.1038/35021046

