# Supplementary material for: Evolutionary rescue in a host–pathogen system results in coexistence not clearance
Source: Evol Appl. 2017 Nov 20;11(5):681–93. doi: 10.1111/eva.12568 (PMC5979755; doi:10.1111/eva.12568)
Supplement: Supplementary file 1 [file EVA-11-681-s001.pdf]

Supplemental Materials

Supplementary materials for "Evolutionary rescue in a host-pathogen system results in coexistence not clearance," by M.R. Christie and C.L. Searle published in *Evolutionary Applications.*

Table S1: Parameter estimates based on previously published literature on anurans in North America.

Parameter	Estimates<br>*Justification or notes*	Values used in simulations<br>*Justification or notes*	References*
K_{adults}	Breeding adults: < 10 to > 8000<br>*There is wide variation in* K_{adults} *among species and populations. It is common for anurans to experience large fluctuations in* K_{adults} *between years.*	50 – 1200	Pechmann *et al.* 1991; Meyer *et al.* 1998
K_{tads}	0 to > 7,500<br>*There is wide variation in* K_{tads} *among species and populations. It is common for anurans to experience large fluctuations in* K_{tads} *between years.*	50 – 5000	Pechmann *et al.* 1991; Blaustein *et al.* 1994
Eggs laid per breeding pair	8 *(Rana grylio)* – 45, 000 *(Bufo cognatus)*<br>*Most species lay 100 – 3000 eggs each year and there is wide variation between and within species.*	100<br>*This value was chosen to be logistically tractable in our model. There is high mortality between the egg and metamorph stage such that our model mimics the high variance in reproductive success observed in the wild.*	Herreid & Kinney 1966; Calef 1973, Data from Bancroft *et al.* 2011
Percent mortality after *Bd* infection	0 – 100%<br>*Mortality rates after exposure to Bd in laboratory experiments.*	0 – 100%<br>Bd-*related mortality was present in our model as* GSM *with a range of 0 – 1.*	Reviewed in Kilpatrick *et al.* 2010; see Table S1 in this publication
Time to metamorphosis	Less than 4 months (many species) – 3 years (*Rana muscosa, R. catesbeiana, R. sierrae*)<br>*The majority of North American anurans complete metamorphosis in less than 12 months.*	1 summer<br>*In our model, all individuals completed metamorphosis each year.*	Data from Bancroft *et al.* 2011

*References are listed at the end of this document (supplemental materials).

Table S2: Parameter values for all simulations.

**Manipulation**	**Parameter**	**Values**
Fixed within-population mortality	*GSM**	0, 0.1, 0.2, 0.3, 0.4, 0.5, 0.6, 0.7, 0.8, 0.9, 0.99
	K_{adults}	100, 200, 300 adults
	K_{tads}	1000 tadpoles
	Transmission	Low (0.2), moderate (0.3), high (0.4)†
Mixed within-population mortality	*GSM* distribution	uniform, normal, log-normal
	GSM range	0.0-1.0, 0.1-0.9, 0.2-0.8, 0.3-0.7, 0.4-0.6, 0.49-0.51
	GSM mean	0.5
	K_{adults}	200 adults
	K_{tads}	1000 tadpoles
	Transmission	moderate (0.3)
Exploring parameter space	Number of *GSM* values‡	1, 2, 3, 4, 5, 6, 7, 8, 9, 10, 15, 20, 30
	K_{adults}	50, 200, 400, 600, 800, 1000, 1200 adults
	K_{tads}	50, 1000, 2000, 3000, 4000, 5000 tadpoles
	Transmission	0.25, 0.5, 0.75, 1.0, 1.25, 1.5, 1.75, 2.0, 2.25, 2.5, 2.75, 3.0, 3.25

**GSM* = genotype specific mortality, which is the probability that an individual dies each year if infected with *Bd*.

†Transmission values are the distance required (in meters) between a susceptible and infected individual for transmission to occur for a 100x100 meter environment, though results would scale irrespective of the unit of distance measurement employed.

‡*GSM* values were selected randomly from a uniform distribution ranging from 0-1.

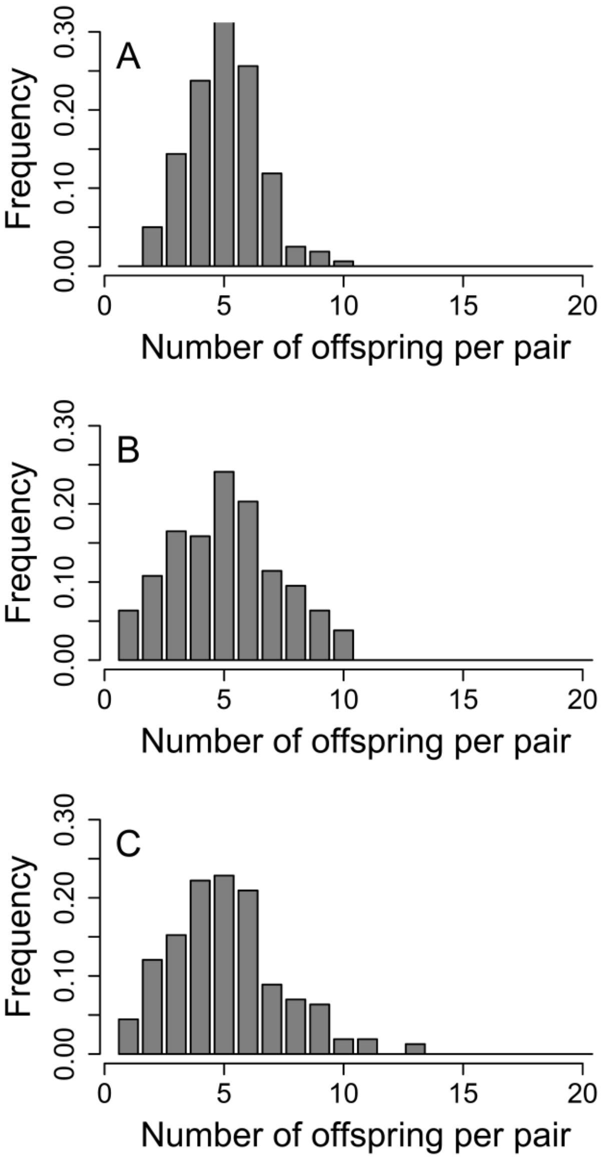

Figure S1: Average number of offspring per pair of breeding adults measured after density-dependent mortality of the tadpoles, but before *Bd*-associated mortality or adult mortality. Notice that there is relatively high variance in reproductive success for each pair and that the average number of offspring does not substantially change if each pair produces (A) 10 offspring, (B) 100 offspring (default value), or (c) 1000 offspring. As offspring continue to mature, the means of these distributions shift towards the left and the distributions skew further to the right as fewer offspring from each breeding pair survive to reach the adult stage.

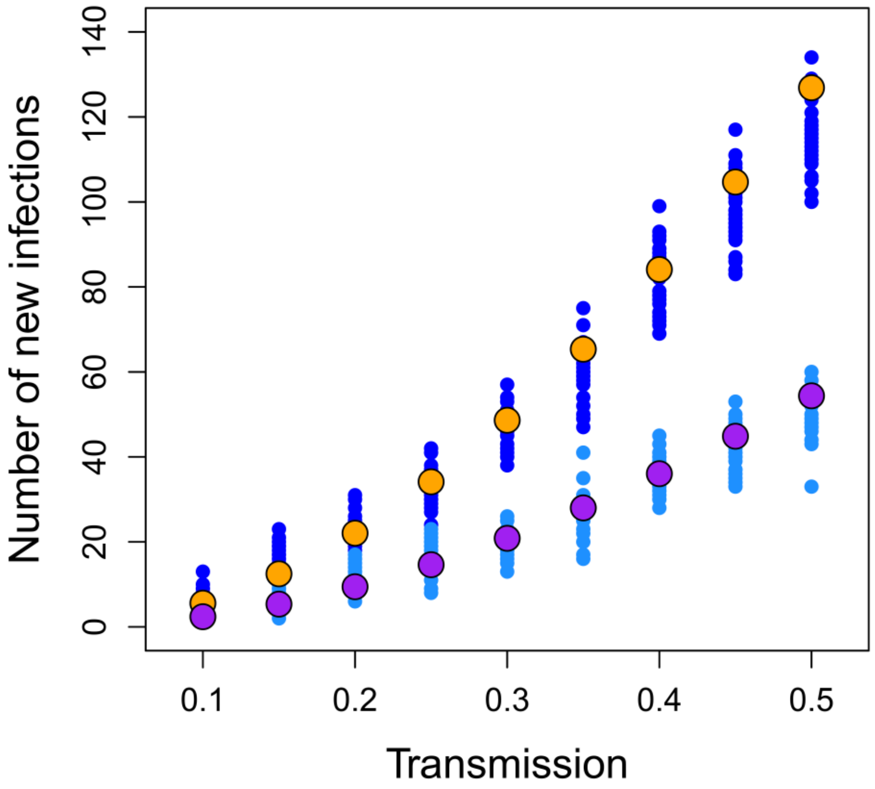

Figure S2: Comparison between the number of new infections using a spatially-explicit, individually-based approach (small circles; see Methods in the main text) and a probability-based approach (large circles). For the probability-based approach the number of newly infected individuals was calculated as:

$$\gamma = 1 - \left(\frac{1 - m}{x}\right)^t$$

where *m* equals the number of cells surrounding an infected individual that contain the pathogen (calculated from the transmission threshold), *x* equals the total number of cells in the pond (here $= 10^8$), and *t* equals the total number of times an individual moved in the pond during a single season (here = 2000). To obtain the total number of new infections γ was multiplied by the total number of hosts. For both approaches, a single individual was introduced into a population and the total number of newly infected individuals was calculated for 30 replicates for each level of transmission. No variation occurred with the probability-based approach for population sizes of 500 (orange circles) and 100 (purple circles). The spatially-explicit approach introduced variation for n=500 (dark blue circles) and n=100 (light blue circles) and overlapped with the probability-based estimates. We consider the added variation among replicates to be more realistic, but such variation could also be included with a probability-based approach. The main point of the figure is to illustrate that either approach to modeling transmission would provide nearly identical results.

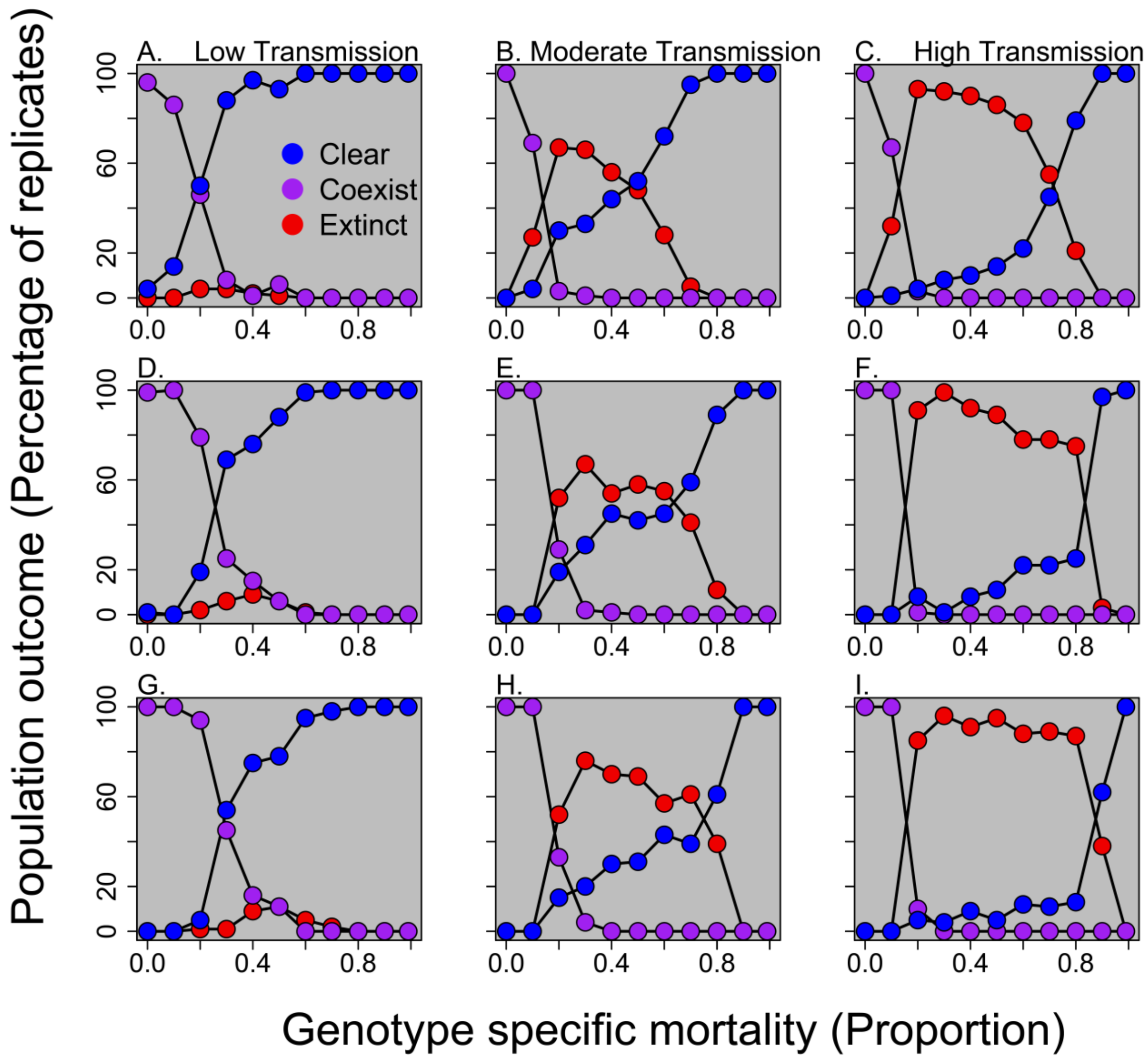

Figure S3: Population outcomes when genotype specific mortality (*GSM*) is fixed within a population, such that all individuals have the same level of mortality if infected. For each value of genotype-specific mortality we performed 100 replicate simulations and recorded the population outcome. Blue points represent the proportion of simulations that cleared infection, purple points represent the proportion of host populations that coexisted with *Bd*, and red points represent the proportion of host populations that went extinct. Columns represent different levels of transmission; low (0.2) shown in A, D, and G, moderate (0.3) shown in B, E, and H, and high (0.4) shown in C, F, and I. Rows indicate different adult carrying capacities (K_{adults}) where in the top row K_{adults} = 100 (A-C), middle row K_{adults} = 200 (D-F; default) and bottom row K_{adults} = 300 (G-I). Tadpole carrying capacity was 1000 for all simulations. Panels D, E, and F where K_{adults} = 200, are the same as those in Figure 2 in the main text.

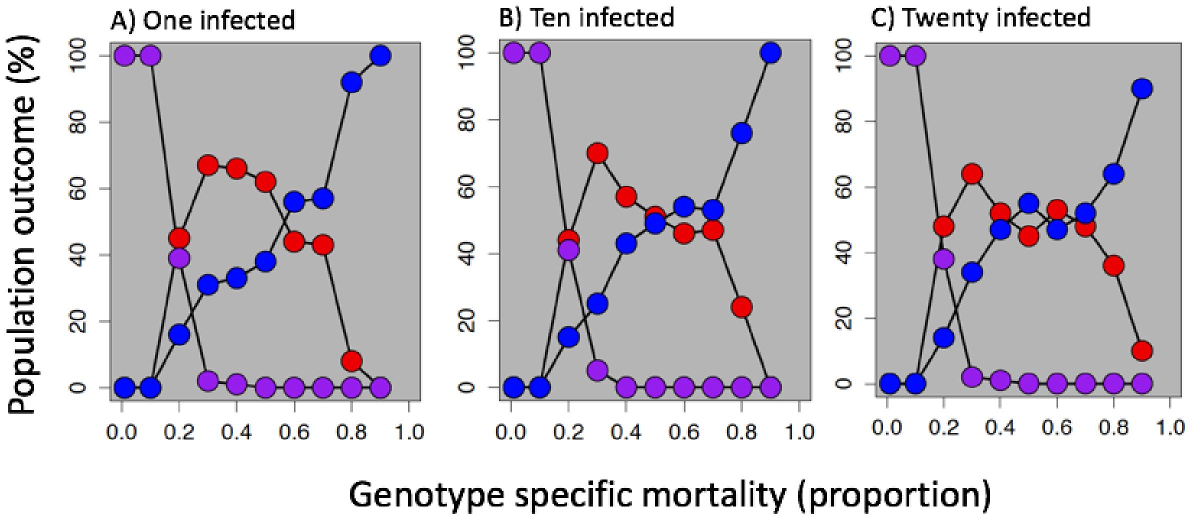

Figure S4: Effects of increasing the number of infected individuals that introduce *Bd* into an unexposed system. For results presented in the main text, we only introduced a single infected adult into each system. Here, we introduced one infected individual (A, similar to Fig 2B main text), ten infected individuals (B), or twenty infected individuals (C). For each value of GSM, we performed 100 replicate simulations. Blue points represent the proportion of host populations that cleared infection, purple points the proportion of host populations that coexisted with *Bd*, and red points the proportion of host populations that went extinct. All populations had a carrying capacity of 200 adults and 1000 tadpoles with moderate transmission and a fixed GSM (same parameters as Figure 2B). Intermediate levels of genotype specific mortality result in a greater proportion of extinctions in all cases. In general, the effect of changing the number of introduced infected hosts was small, but we did see slightly higher rates of extinction at high GSM values when twenty individuals were introduced (panel C) in comparison to when one individual was introduced (panel A).

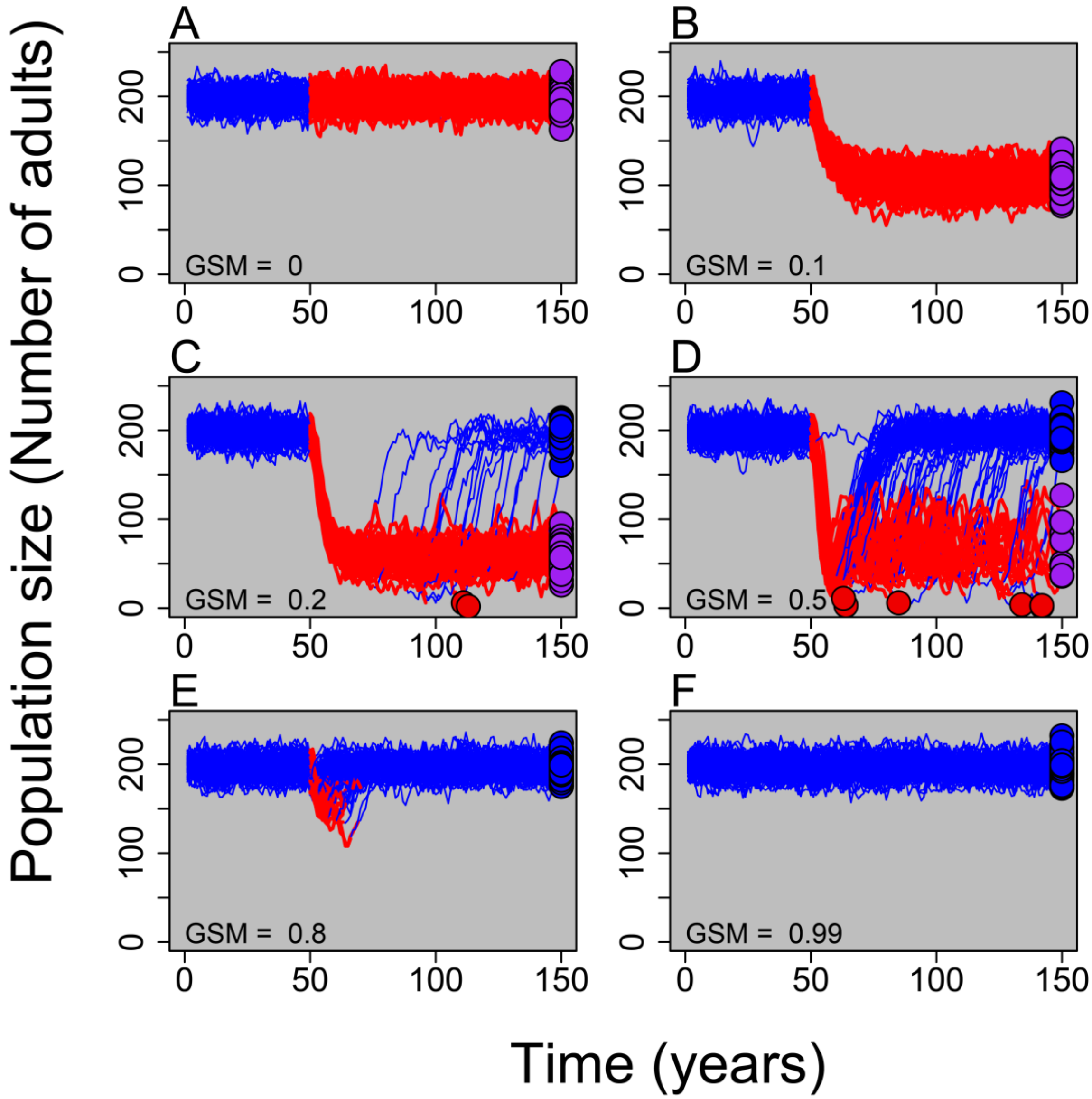

Figure S5: Amphibian host population size (number of adults) through time for 6 different values of genotype specific mortality (*GSM*; indicated in the bottom left of each panel) under low transmission conditions (0.2 m). Each line represents a single model run, of which there were 100 per panel. *Bd* was introduced into the population in year 50. Blue lines represent uninfected host populations while red lines represent populations with at least one infected individual. Dots represent the population outcome where red points (jittered) show host populations that went extinct, blue points show host populations that cleared infection, and purple points host illustrate populations that coexisted with *Bd*. Model parameters were set with K_{adults} = 200 and K_{tads} = 1000 (same as in Fig. 2A in the main text). Fewer populations went extinct at these lower levels of transmission.

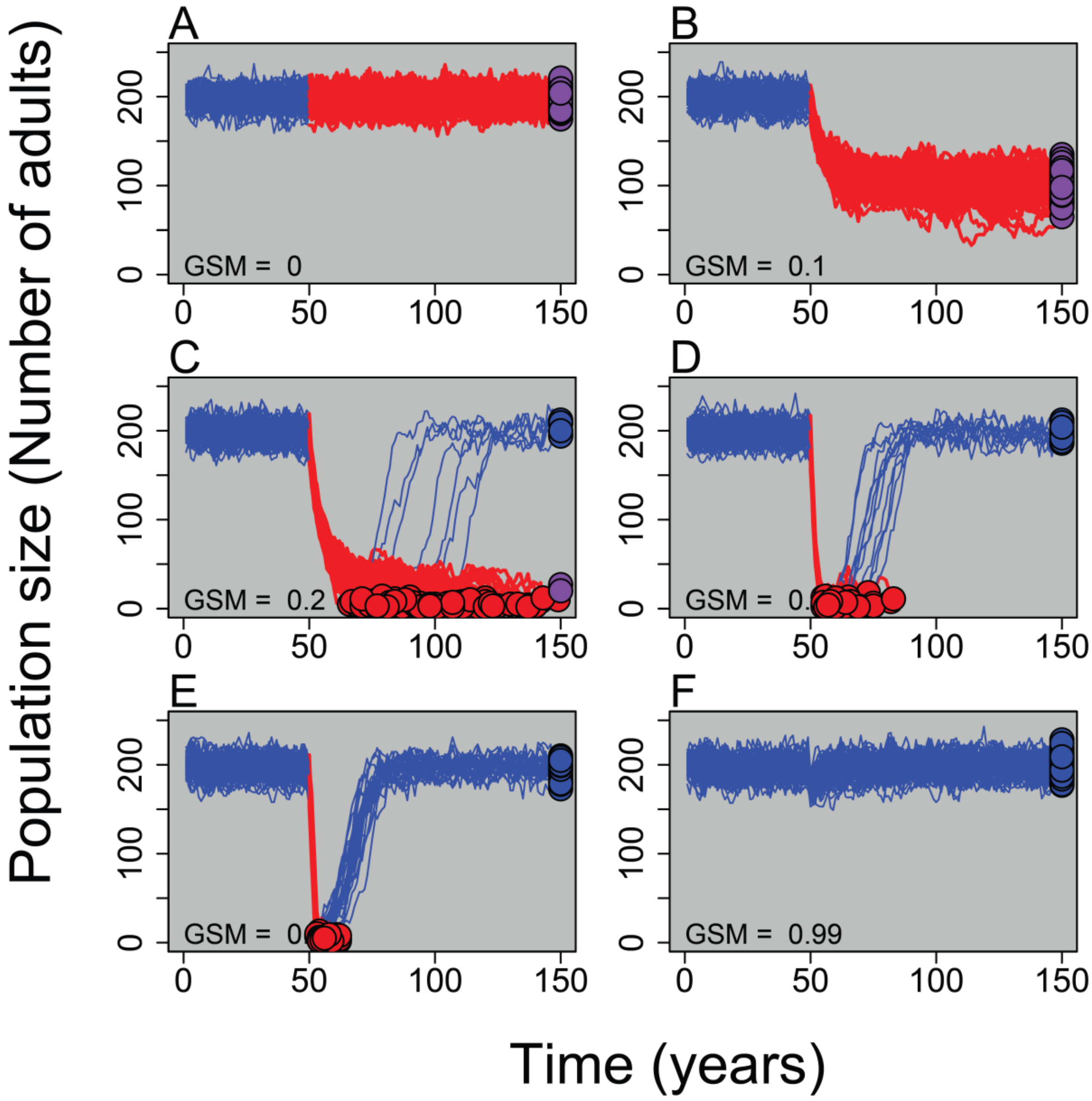

Figure S6: Amphibian host population size (number of adults) through time for 6 different values of genotype specific mortality (*GSM*; indicated in the bottom left of each panel) under high transmission conditions (0.4 m). Each line represents a single model run, of which there were 100 per panel. *Bd* was introduced into the population in year 50. Blue lines represent uninfected host populations while red lines represent populations with at least one infected individual. Dots represent the population outcome where red points (jittered) show host populations that went extinct, blue points show host populations that cleared infection, and purple points host illustrate populations that coexisted with *Bd*. Model parameters were set with K_{adults} = 200 and K_{tads} = 1000 (same as in Fig. 2C in the main text). More populations went extinct at these higher levels of transmission.

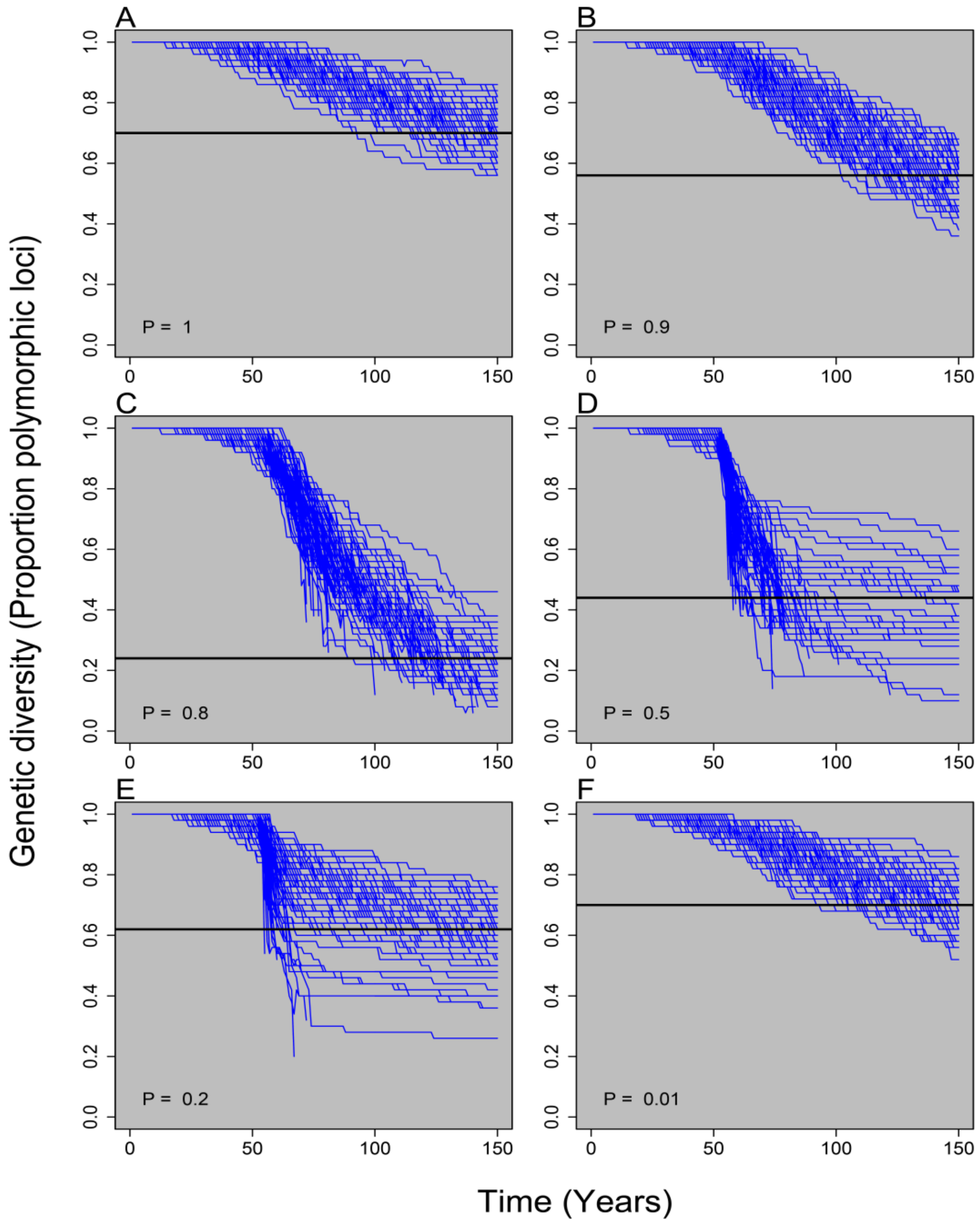

Figure S7: Genetic diversity through time for 6 different values of genotype specific mortality (*GSM*; indicated in the bottom left of each panel). Each line represents a single model run, of which there were 100 per panel. *Bd* was introduced into the population at year 50. Genetic diversity was measured as the proportion of polymorphic loci (*i.e.,* the proportion of loci with more than 1 allele). The solid horizontal line in each panel indicates the median proportion of polymorphic loci in populations that persisted for all 150 years. The populations illustrated in this figure are the same as those illustrated in Fig. 3 & 4 of the main text and the results are similar to when heterozygosity was used as the response variable (Fig. 4).

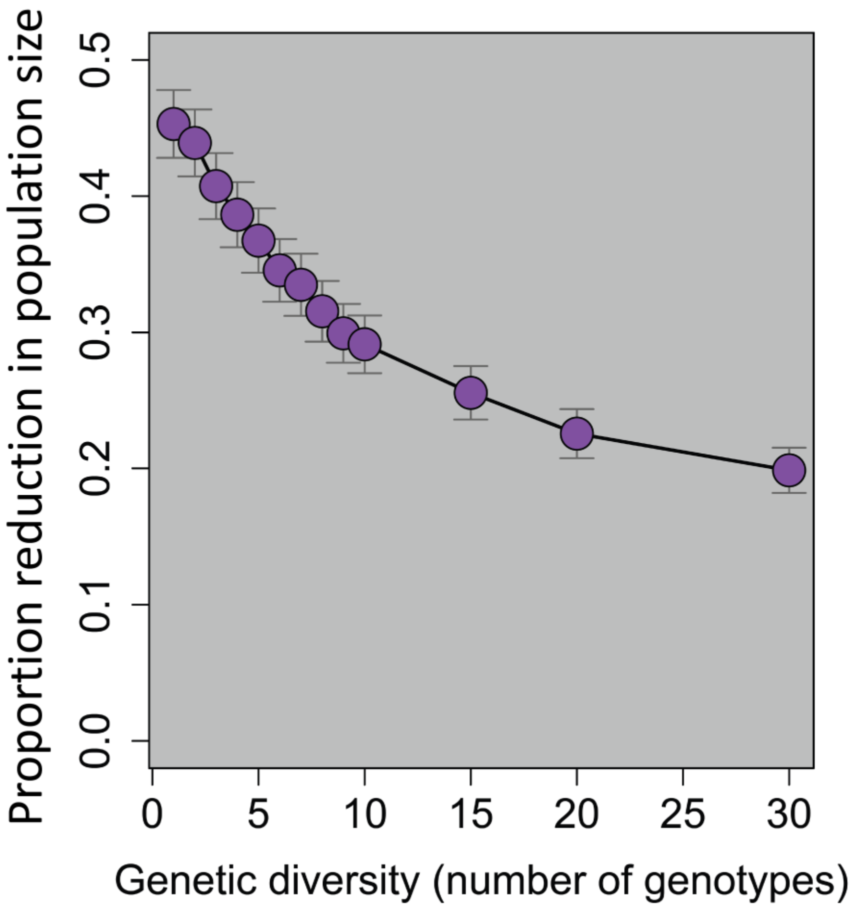

Figure S8: The proportional reduction in adult host population size across levels of adaptive host genetic diversity (where genotypes were selected randomly). A larger value indicates that the host population was reduced to a greater degree in the presence of *Bd*. As genetic diversity increases, the negative effects of *Bd* on host population size decreases. These populations represent all populations that were in the coexist category in figure 6A of the main text.

References:

Bancroft, B.A., Han, B.A., Searle, C.L., Biga, L.M., Olson, D.H., Kats, L.B., Lawler, J.J., Blaustein, A.R. (2011). Species-level correlates of susceptibility to the pathogenic amphibian fungus *Batrachochytrium dendrobatidis* in the United States. *Biodiversity and Conservation*, 20, 1911-1920.

Blaustein, A.R., Wake, D.B. & Sousa, W.P. (1994). Amphibian declines - judging stability, persistence, and susceptibility of populations to local and global extinctions. *Conservation Biology*, 8, 60-71.

Calef, G.W. (1973). Natural mortality of tadpoles in a population of *Rana aurora*. *Ecology*, 54, 741-758.

Herreid, C.F. & Kinney, S. (1966). Survival of Alaskan woodfrog (*Rana sylvatica*) larvae. *Ecology*, 47, 1039-1041.

Kilpatrick, A.M., Briggs, C.J. & Daszak, P. (2010). The ecology and impact of chytridiomycosis: an emerging disease of amphibians. *Trends in Ecology & Evolution*, 25, 109-118.

Meyer, A.H., Schmidt, B.R. & Grossenbacher, K. (1998). Analysis of three amphibian populations with quarter-century long time-series. *Proceedings of the Royal Society B: Biological Sciences*, 265, 523-528.

Pechmann, J.H.K., Scott, D.E., Semlitsch, R.D., Caldwell, J.P., Vitt, L.J. & Gibbons, J.W. (1991). Declining amphibian populations - the problem of separating human impacts from natural fluctuations. *Science*, 253, 892-895.
